# Ethical and practical considerations arising from community consultation on implementing controlled human infection studies using *Schistosoma mansoni* in Uganda

**DOI:** 10.1080/11287462.2022.2091503

**Published:** 2022-07-04

**Authors:** Moses Egesa, Agnes Ssali, Edward Tumwesige, Moses Kizza, Emmanuella Driciru, Fiona Luboga, Meta Roestenberg, Janet Seeley, Alison M. Elliott

**Affiliations:** a Immunomodulation and Vaccines Programme, MRC/UVRI and LSHTM Uganda Research Unit, Entebbe, Uganda; b Uganda Virus Research Institute, Entebbe, Uganda; c Department of Infection Biology, London School of Hygiene and Tropical Medicine, London, UK; d Social Aspects of Health Across the Life-Course Programme, MRC/UVRI and LSHTM Uganda Research Unit, Entebbe, Uganda; e Department of Parasitology, Leiden University Medical Center, Leiden, The Netherlands; f Department of Global Health and Development, London School of Hygiene and Tropical Medicine, London, UK; g Department of Clinical Research, London School of Hygiene and Tropical Medicine, London, UK

**Keywords:** Controlled human infection, informed consent, perceptions, schistosomiasis, endemic

## Abstract

Issues related to controlled human infection studies using *Schistosoma mansoni* (CHI-S) were explored to ensure the ethical and voluntary participation of potential CHI-S volunteers in an endemic setting in Uganda. We invited volunteers from a fishing community and a tertiary education community to guide the development of informed consent procedures. Consultative group discussions were held to modify educational materials on schistosomiasis, vaccines and the CHI-S model and similar discussions were held with a test group. With both groups, a mock consent process was conducted. Fourteen in-depth key informant interviews and three group discussions were held to explore perceptions towards participating in a CHI-S. Most of the participants had not heard of the CHI-S. Willingness to take part depended on understanding the study procedures and the consenting process. Close social networks were key in deciding to take part. The worry of adverse effects was cited as a possible hindrance to taking part. Volunteer time compensation was unclear for a CHI-S. Potential volunteers in these communities are willing to take part in a CHI-S. Community engagement is needed to build trust and time must be taken to share study procedures and ensure understanding of key messages.

## Introduction

Schistosomiasis affects approximately 200 million people worldwide, with a loss of 1.4 million disability-adjusted life years (Kyu et al., 2018). The greatest disease burden is in sub-Saharan Africa (Mutapi et al., 2017), a third of which is caused by *Schistosoma mansoni* (*Sm*) (van der Werf et al., 2003). An effective vaccine would promote immunity to reinfection and is therefore urgently needed (Alsallaq et al., 2017). Controlled human infections (CHI) can accelerate identification of the most promising vaccine candidates (Roestenberg et al., 2018; Sauerwein et al., 2011; Waddington et al., 2014) and immune correlates of protection (Haney et al., 2018). Details of the scientific basis of CHI with *S. mansoni* (CHI-S) are summarised in supplementary material S1. CHI studies need to be undertaken with particular attention to ethical considerations, which revolve around the core issue of the potential to cause harm through deliberate infection of healthy subjects (Hausman, 2021). A team at the Leiden University Medical Center in the Netherlands safely conducted the first controlled human infection with *S. mansoni* CHI-S (Langenberg et al., 2020). We now propose to establish the CHI-S model in Uganda, where *S. mansoni* is endemic. Implementation in an endemic setting is key for conditions, such as schistosomiasis, where prior exposure to the same pathogen (*in utero*, through maternal infection, or directly, through exposure in the early months and years of life) can have important impacts on the immune response to infection and to candidate vaccines. As well, unrelated environmental exposures can modulate vaccine responses in rural low-income settings (Driciru et al., 2021). However, implementation in such settings intensifies the need for careful attention to ethical issues, including benefits and risks to participants, informed consent, payment to participants and community engagement (Jamrozik & Selgelid, 2020a; Selgelid & Jamrozik, 2018).

A workshop on CHI-S studies in Entebbe in Uganda in 2017 which included policy-makers, research regulatory key personnel, community members and researchers developed a roadmap for CHI-S implementation in Uganda (Elliott et al., 2018). The workshop identified the ethical imperative to engage with Ugandan communities from whom CHI-S volunteers might be recruited and to develop materials and consent forms with their participation to ensure that the communities, and the potential CHI-S volunteers, would understand fully the nature of CHI-S and its risks, as well as its societal benefits. Additionally, it was important to determine the feasibility and acceptability of participation in CHI-S for members of the target communities, especially where exposure to infection (through activities in water bodies harbouring the intermediate snail host, such as Lake Victoria) is essential to economic activities such as fishing. In this paper, we share our findings in a study, where we engaged with Ugandan communities from which CHI-S volunteers might, in future, be recruited. Our objectives were to obtain community input into the development of informed consent procedures and to explore community perceptions of the CHI-S model, including risks, benefits, participant selection, feasibility and attitudes to remuneration.

## Materials and methods

### Study communities

For future *S. mansoni* vaccine studies in Uganda, it will be important to enrol members of populations both unexposed and previously-exposed to *S. mansoni* to compare vaccine responses. For this work, we engaged adults from a university community, and a high-transmission fishing community on the Entebbe peninsula. The target communities selected were within easy reach of the clinical and laboratory facilities at the Uganda Virus Research Institute (UVRI), the proposed host institution for future CHI-S studies.

The university community comprises a well-educated population from across Uganda and is expected to include individuals previously unexposed or minimally exposed to *S. mansoni*. This community was proposed based on experience from Kenyan colleagues for initial studies on CHI models for malaria, where a university community in Nairobi was found to be appropriate (Hodgson et al., 2014). However, a CHI stakeholders’ meeting in Uganda identified ethical concerns about students who, although adults, are still dependents and suggested the need for parental involvement in the recruitment of undergraduate students, since they are commonly still dependents. Therefore, for this work, we consulted across the more independent spectrum of the university community. We considered university staff including administrators and teachers as well as post-graduate (Masters and PhD) students and mature-entry undergraduates, among potentially eligible candidates. Mature-entry undergraduates are those who join university years after leaving secondary school, and are mainly self-funded students.

A high *S. mansoni* transmission fishing community was selected on the shores of the Entebbe peninsula. This was considered more appropriate than island communities for future CHI-S studies because of proximity to the clinic and laboratories at the UVRI and because access to safe water supplies and sanitation facilities is available – rendering avoidance of re-infection during CHI-S studies possible.

### Selection and recruitment of participants

**Figure 1.**
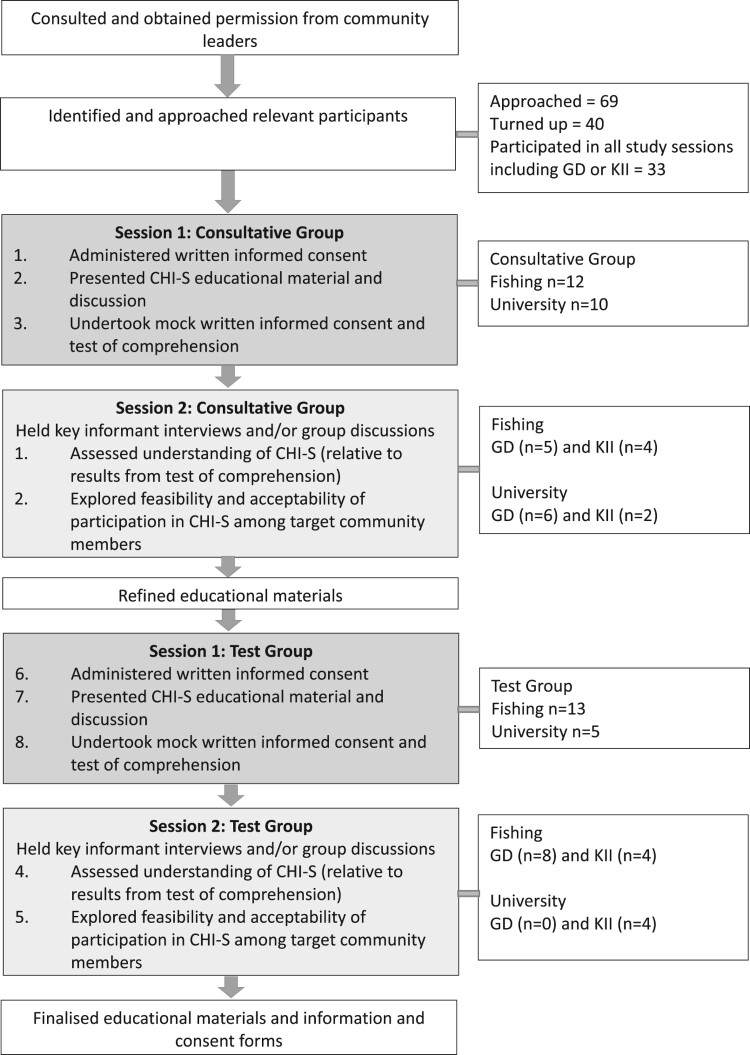
Study flow. The activities were conducted in each of the two target communities.

The study was conducted between July and November 2019. The study flow is set out in Figure 1. “Participant” refers to the community member who took part in this descriptive study and “volunteer” is used to refer to a community member who will take part in the future CHI-S.

First, we approached the administrative leaders of the university, and the community leaders in the fishing villages, explained this study and obtained their permission to work within their communities. Having obtained permission, we recruited two groups of participants aged 18–50 years in each setting. The first group in each setting was a “Consultative Group” and the second, a “Test Group”. The participants were purposively selected by the peer leaders in the university and fishing communities. Participants from the university were selected due to their leadership in faculties and their years of study. The community participants were influential fishermen, young men and women leading in the community and those who were engaged in some form of business near the lake shore. Having identified potential participants, we explained the consultative nature of this study and that we were seeking their input in developing future work towards vaccines for schistosomiasis. Written informed consent for participation in the CHI-S preparatory study was obtained before further study activities were undertaken. The participants were asked for their verbal consent to record the interviews and the group discussions. The timing of the meetings and interviews was agreed upon between the research team and the participants. Personal characteristics of consenting participants were recorded, including age, sex, occupation and education level, and both current and previous places of residence (since this has implications for *S. mansoni* exposure).

### Developing educationally-appropriate materials

As a first step to involve the community, consented participants were invited to discuss and to make suggestions on educational materials. The educational materials were designed by the research team and were based on materials commonly used for health education by the Uganda Ministry of Health's Vector Control Division. The materials were developed for the basic reading level to ease understanding of the CHI-S, translated into Luganda (the main local language commonly used in these communities) and were read by the participants on the first day of consultations. The educational materials were brochures of simplified content describing schistosomiasis transmission, control and prevention approaches; vaccine testing and approaches; and the CHI-S model, dose finding, expectations, risks and benefits (supplementary material S2). The primary risks of CHI-S procedure described in the educational materials were symptoms that may be seen in CHI-S volunteers. These were rash, allergy-like symptoms, fever and cough. In the same materials, the participants were informed that the expected symptoms can be treated and a doctor will be available to the volunteers via telephone 24 hours. The materials were distributed to the participants on the day of the session to read them together with the research team. At the end of the session, the participants were asked to consult with family and relatives for a week.

Suggestions made after a week were used to modify materials on the CHI-S model brochure, describing the technical processes involved in lay terms, explaining potential adverse effects of CHI-S upon participants, and describing the requirements in terms of time and procedures expected from future CHI-S volunteers.

For university community participants, a power-point presentation of materials in English was made and written explanations on brochures in English shared. For the fishing communities (where power-point presentations were unfamiliar and impractical), the educational materials were translated into Luganda, the local language to accommodate those who could not read English, but the presentation content was exactly the same as for the university community.

All participants attended the first meeting for the presentation and modification of materials, then they discussed through a mock CHI-S volunteer information sheet and consent forms adapted from colleagues at the Leiden University Medical Center in the Netherlands. The information sheet and consent form were translated into Luganda (the commonly spoken language in the study area). Participants were given 10 multiple-choice questions to test comprehension (supplementary material S3). Each question was given one point for a correct response and each an unanswered question or incorrect answer got a zero-point score. A calculation of a cumulative knowledge score for each question and for each group was taken and summarised in a Table 1.

**Table 1.** Test of comprehension results for the communities (*N* = 40).

Question topic	Fishing community			University community			Total Score per question *N* (%)^a^
	Consultative group *n* = 12	Test group *n* = 13	Score *N* (%)	Consultative group *n* = 10	Test group *n* = 5	Score *N* (%)	
1. Where do we get schistosomiasis from?	10	12	22 (88)	9	5	14 (93)	36 (90)
2. Can schistosomiasis be stopped by minimising contact with infested water?	11	12	23 (92)	9	5	14 (93)	37 (93)
3. Could a vaccine prevent schistosomiasis?	12	11	23 (92)	10	5	15 (100)	38 (95)
4. What are the characteristics of a controlled human infection study?	5	7	12 (48)	7	3	10 (66)	22 (55)
5. Is determining the number of larvae to safely use on volunteers the first step in CHI-S?	10	9	19 (76)	10	4	14 (93)	33 (83)
6. What is involved in testing a vaccine using controlled human infection?	10	6	16 (64)	5	2	7 (47)	23 (58)
7. Why do volunteers come into the clinic every week after infection [CHI-S]?	10	4	14 (56)	3	3	6 (40)	20 (50)
8. Could a controlled human infection with *S. mansoni* cause symptom in volunteers?	12	11	23 (92)	10	4	14 (93)	37 (93)
9. What does a volunteer need to do before he/she can participate in the study?	11	6	17 (68)	9	5	14 (93)	31 (78)
10. Do volunteers personally benefit from participating in CHI?	3	6	9 (36)	4	4	8 (53)	17 (43)
Total score per group, *n* (%)^b^	94 (78)	84 (65)	178 (71)	76 (76)	40 (80)	116 (77)	

^a^ The percentage is the number of participants with correct answers divided by the total number of participants (i.e. 40).

^b^ The percentage is the number of correct answers divided by the possible total of correct answers for each group in each setting.

### Qualitative data collection

One week after the discussion of materials, the participants were randomly selected to either participate in a group discussion (GD) or key informant interview (KII). The one-week interval allowed participants to reflect on what they had heard about CHI-S model, share sample education materials, and to discuss with family and friends, as would be the case in a genuine CHI-S recruitment process before one is enrolled in a trial. A local language (Luganda) topic guide and an English translation of that guide were generated and used for interviews conducted in Luganda and English in the fishing and university community respectively. Key topics discussed are presented in the guide for KII (supplementary material S4) and GD (supplementary material S5).

For the KII, four to six individuals were targeted from each setting. At the university, a lecturer, a postgraduate student, an administrator, and one undergraduate were targeted. In the fishing community two community leaders (a male and a female), two men and two women directly and indirectly involved in fishing were targeted. We chose to include equal numbers of men and women because the roles played in fishing and trading, and in the support structures for the fishing industry (shops, bars and restaurants, homes and lodges) differ by gender. The aim of this selection was to obtain a variety of representation of the two study communities. In the GD, we used scenarios to facilitate the discussion of what taking part in such a trial would mean to volunteers in terms of time commitments and study procedures that would be expected for future CHI-S volunteers.

The semi-structured topic guides to solicit information on how well participants had understood the CHI-S model, included questions that asked about the purpose of CHI-S trial, its risks to volunteers, potential societal benefits, attitudes to the risks involved in CHI-S participation, and explored reactions that participants received when sharing with family and friends, about the planned trial, how educational materials were viewed, and how the consent process could be improved upon, what attitude the participants had to CHI-S – whether they thought it should be implemented in Uganda, whether they would take part and whether they would support their friends and family to take part in such a study.

Other aspects explored included the implications of participation for time off work, studies and daily responsibilities for clinic visits and the feasibility of this; likely costs, or financial losses, that would be incurred; feasibility of avoiding contact with contaminated water during the 12-week CHI-S studies; attitudes to compensation for participation in CHI-S and expectations as to what amount of cash in Uganda Shillings would or should be provided.

### Data management

Data comprised transcripts from recordings of KIIs and GDs. The team members who conducted interviews or group discussions generated a summary of the interview or group discussion immediately on their return to the office, and before the transcription was done, to capture the main issues arising and to note any particular issues arising during the conduct of the data generation (interruptions, demeanour of the participants). This summary was used in the post-interview/group discussion debriefing with the study team.

All written outputs were reviewed by the team lead and returned to the team member responsible for clarification if anything was unclear and to ensure that they conform to the agreed levels of anonymisation. All data were anonymised at the time of transcription with the names of respondents replaced by a unique anonymised participant identifier. Any names of people mentioned, or of respondents, were replaced with initials.

All recordings were erased once transcription and checking of transcripts was complete. The data was uploaded to a password-protected computer and backed up on the MRC/UVRI & LSHTM Uganda Research Unit data server. Transcripts of interviews held in Luganda were transcribed and translated into English for analysis.

### Data analysis

Each transcript of the KIIs and GDs was manually coded using Microsoft Word and the main codes were agreed upon by the research team through open coding approach. The codes followed the key topic questions in the interview guides. The coded data from each transcript were charted into a matrix showing the cases across the matrix. The choice of thematic headings was guided by the core concepts emerging out of the data.

Data were analysed using framework thematic analysis. Framework analysis involves indexing all verbatim text, charting information from each transcript onto a series of thematic matrices (Ritchie et al., 2013). The charting of data onto a matrix helps the researcher to look at both the case across and the theme that are in descriptive columns. The iterative process has the advantage of keeping the context of the data, it is systematic, comprehensive and transparent when agreeing on explanations of the data. The findings are detailed in supplementary material S6**.**

### Ethics statement

We obtained ethical approval from the UVRI Research Ethics Committee (reference number GC/127/19/02/703), London School of Hygiene and Tropical Medicine Observational/Interventions Research Ethics Committee (reference number HS 17135) and the Uganda National Council for Science and Technology (reference number HS 2564). For this study, participants contributed their time and opinions. The main inconvenience to the participants was time away from work/study. The consultative sessions took approximately two and a half hours and the KIIs and GDs each took about one hour. These were scheduled with consideration for participants’ convenience. The participants were given 25,000 Uganda Shillings (about USD 7) to reimburse their transport expenses and to compensate for the time spent to participate in the study. All participants provided written informed consent before taking part in this study.

## Results

In the results we first present the feedback from the meetings to discuss the educational materials, then we highlight identified themes from the interviews and the group discussions that reflect the practical and ethical implications of conducting a CHI-S study.

### Feedback on and comprehension of the educational materials

The flyers (with educational materials) were found to be easy to carry. The materials were quite informative and could help one understand the CHI-S model well, however, there was a suggestion by one participant for the need to present the information in a booklet form or a poster form so that everything can be seen at the same time. One participant from the fishing community reported that they still required someone to explain the pictures to them in person and not only rely on the pamphlets in order to understand the pictures.

Minor changes to the educational materials were recommended by both the consultative and test group. Some participants commented that the font size of words needed to be made bigger, and some of the images needed to be bigger. Other comments from the university community on the materials included the following:

[The research team should] specify the type of snails since not all snails transmit Bilharzia … use a statement like “kill snails that transmit Bilharzia”

The pictures are too small, words seem [to be] more than pictures … . [the research team] could increase size of the pictures and reduce the font of the words since pictures communicate better than words

[The] picture does not depict “stunted growth” but rather distended abdomen. It would be better to have two pictures: one of a child depicting stunted growth and one of an adult depicting distended abdomen

An additional picture of a person not vaccinated getting infected [would] clearly contrast the picture of the vaccinated person who is protected from infection. This will clearly communicate the significance of a vaccine to any person reading the educational material without explanation

On the other hand, participants from the fishing community mainly recommended grammatic changes to the educational materials that had been translated to Luganda.

The tests of comprehension showed that the questions on schistosomiasis and vaccines (Questions 1–3) were responded to more correctly compared to the questions about the CHI-S model (Questions 4–10) (Table 1). However, the participants clearly understood that the CHI-S may cause symptoms (Question 8).

No clear difference in the results of the tests of comprehension were seen between the consultative group and the test group, or between the university and the fishing communities.

### Social demographic characteristics of participants

Three GDs (two with participants in the consultative group and one GD with participants in the Test group) were held with a total of 19 participants in the two communities. The second GD for the Test group in the University community could not reach quorum and was not conducted. We held KIIs with 14 individuals in this qualitative study, six in the university community, and eight in the fishing community. The socio-demographic characteristics of these participants are summarised in Table A1. Gender and age in the table were considered but had no apparent bearing on the discussions and interviews.

**Table A1.** Sociodemographic characteristics of the participants in GDs and KIIs (*N* = 33).

	University community		Fishing community	
Characteristic	Consultative group (*n* = 7)	Test group (*n* = 4)	Consultative group (*n* = 10)	Test group (*n* = 12)
Sex				
Females (*n*)	3	2	6	5
Age (range)				
18–35	5	1	7	4
36–50	1	2	2	7
Over 50	1	1	1	1

The themes identified in the GD and KII findings; highlight the key aspects which may influence volunteer participation. These include knowledge on the main topic of schistosomiasis, knowledge about vaccines in general, understanding of CHI-S, adherence to CHI-S procedures, potential benefits of CHI-S, concerns about CHI-S, compensation for volunteering in the study. In addition, there was a theme on volunteer perception of the informed consent documentation, and willingness to take part in CHI-S studies.

### Knowledge about schistosomiasis

The majority of participants mentioned that they had heard about schistosomiasis (more commonly called Bilharzia among the public in Ugandan settings). This was especially true for the ones from the fishing community who revealed that they had seen people who had suffered from schistosomiasis. On the other hand, those at the university rarely saw people suffering from schistosomiasis. During the main discussions, participants defined the typical schistosomiasis symptom as the presence of a swollen abdomen of those who are sick.

Most participants from the fishing community mentioned that praziquantel given for the treatment had side effects for instance diarrhoea and vomiting, especially in children. One male participant reported this about praziquantel treatment:

There is no way you [research team] can just come and say let us give children the tablets yet for me I faced a lot of challenges and I accept. [For example,] when you take those tablets, you have to [be] closer to the toilet because every time you want to go to the toilet for the whole day. Male participant, KII, fishing community.

The risk factor for schistosomiasis mentioned, especially by the fishing community participants, was people defecating in the lake or at lake shores. Snails were only mentioned as a transmitting vector for schistosomiasis in the GD held at the university. The respondents reported that the people who suffer most from schistosomiasis are those who get in contact with lake water and these include fishermen, casual workers at the lake who lift people and luggage into and from the boats, children who swim or collect water from the lake and women who do laundry in the lake.

Participants in GDs at both study sites considered schistosomiasis as one of the major public health conditions, besides HIV and malaria, affecting their community. One participant noted that because worms responsible for causing schistosomiasis are present in the fresh water body where they earn their livelihood or collect water, she would participate in a CHI-S focusing on the development of a vaccine for schistosomiasis.

I would agree to participate so we can get a vaccine for Bilharzia. Bilharzia lives in the lake, there are worms in the water and when you step in this water they enter you. Female participant, GD, fishing community.

Participants from the university community expressed advanced knowledge about schistosomiasis, they said much of the knowledge they had about schistosomiasis was acquired in school. One participant did not consider it as a serious disease in their community as compared to malaria:

I have not heard of a person dying of Bilharzia but I have heard and seen many people dying of malaria. Male participant, KII, university community.

### Knowledge about vaccines

Participants across the two study sites demonstrated clear knowledge about vaccines, and said they would welcome a vaccine for schistosomiasis if it became available:

Me as a resident and a leader … I would feel happy because we will happily live in our community without such a disease like Bilharzia. Male participant, KII, fishing community.

A vaccine prevents a person from getting a disease. For example, with a Bilharzia vaccine, once you get it you cannot get Bilharzia even when you go in the lake. Female participant, KII, fishing community.

All the participants mentioned that vaccines are welcome because the current treatment for schistosomiasis, praziquantel, has many side effects and for that reason people refuse to take it. Many participants from the fishing community, while discussing the proposed vaccine, related it to vaccines commonly given to children such as the measles vaccine. A few participants mentioned the usefulness of a vaccine as “helping the body to be resistant to Bilharzia”. University participants were equally in support of development of a vaccine for schistosomiasis:

I think it is a very good idea just like the way they have developed vaccines for measles in children, vaccines for polio and other immunizable diseases, it would be very good to develop a vaccine for Bilharzia as well. Male participant, KII, university community.

One participant said: “prevention is better than cure” and compared a vaccine to building a fence so that one is not attacked by anyone who wants to come onto one's residential property. Participants noted that other interventions, especially giving treatment, had failed to stop the spread and transmission of schistosomiasis and therefore developing a vaccine for schistosomiasis would be useful.

We need a vaccine, Bilharzia is deadly and some people do not know that they have Bilharzia and some people are aware but they don't think it kills so I think it is wise and we should consider introducing this model. Male participant, GD, university community.

### Understanding of CHI-S

At the introduction of the topic, three participants from the university community reported they had heard about what CHI is and how it is conducted. During the first sessions in the fishing community when the CHI-S materials were presented, all participants seemed baffled with what was being presented. In the follow-up individual and group discussions, all the participants recalled most aspects that had been mentioned about the model.

A person will be exposed to male parasites that cause Bilharzia and he will be monitored and after he will be treated against the parasites he is exposed to and it is said that this person may or may not get side effects from the parasites he has been exposed to. Female participant, KII, fishing community.

Several participants during both the KIIs and GDs said that the worms would be introduced by the use of an injection. However, participants, from both the fishing and university community, supported each other to correct what was not well understood by peers during the GDs. For example, they informed their peers that the worms are put on the skin and penetrate as they would naturally but are not injected inside the individual.

… parasites are introduced on your skin whereby they pierce [penetrate] the skin and enter on their own. Male participant, GD, university community.

In four of the KIIs, participants mentioned male worms, the rest left it quite open and one said they could not recall whether male or female worms are used in the CHI-S. The age group limit (18–45 years) for being part of the future CHI study was mentioned by three individuals in three KIIs and in all the GDs. All participants recalled that the volunteer for CHI-S would need to be healthy, this was usually expressed in general terms although a few mentioned that one needed to be HIV negative. There was also mentioned by at least three individuals and in one GD that pregnant women were not to be included in the model study. And a few participants reported that if one had some illness such as diabetes they would not be included in the study. Two participants mentioned (correctly) that a volunteer would be given 10 worms to start with. A female participant, when asked what she recalled about CHI-S information shared with her, said;

I don't remember well but you [the research team] said that it is voluntary and that they put in you worms and they monitor you and that you have to be aged 18 years and above. I also remember that they cannot put worms in you when you are pregnant and when you are HIV positive. Generally, you said a person has to be healthy. Female participant, KII, fishing community.

### Adherence to CHI-S procedures

All the participants in the fishing community mentioned that the fishing activities may not be stopped because they are the main source of livelihood for most people at the lake. They mentioned that it was difficult for women who sell food in restaurants to stay away from the lake unless tap water is introduced near their workplaces. The GD participants in the fishing community suggested that the water for domestic use needed to be provided for free from the municipal taps if women were to avoid the lake. In some places where there is tap water, some participants mentioned that they were not willing to wait for long in queues at the taps yet the lake water is easy to access.

Some participants felt it may not be feasible to include fishermen in this study. They said it is difficult for a fisherman to keep away from the lake for the study period because he earns from the lake to sustain his family.

[The lake] is not only their [fishermen's] source of income and livelihood, the lake is part of them, the lake is their life, they sleep there and everything is done there so convincing them is not easy. Male participant, GD, university community.

For me not to go to the lake, something special has to be done. Us here we [fishermen] are used to earning everyday. Male participant, GD, fishing community.

Some participants suggested that people who are not earning from working at the lake should be invited to volunteer in the proposed study.

### Potential benefits of CHI-S

Participants mentioned benefits were likely to include the safety of families from acquiring schistosomiasis, schistosomiasis will eventually reduce in their community and the societal benefit with less sick people. The majority of participants noted that CHI-S will lead to the development of a vaccine which they said will be more beneficial for the society than for individual volunteers.

The benefit is that Bilharzia will be reduced in our area to the extent that it will not be common like it is now. Female participant, KII, fishing community.

Of course, [by] participating you are helping the entire society. You are trying to test a vaccine and the benefits are on a bigger magnitude because you are participating in developing a vaccine that can be administered to people across the country. Male participant, KII, university community.

A few participants mentioned that there were other benefits of CHI beyond the societal benefit. Such other benefits included improving medical research in Uganda according to the university community while monetary benefits, in terms of compensation for participating in the study, were mainly discussed by the fishing community who perceive the lake as their main source of livelihood and would be affected if they are deterred from accessing the lake.

### Concerns and suggestions about participating about CHI-S

Participants highlighted some concerns regarding CHI-S. The most emphasised concern was the safety of the participating volunteers.

It is hard to convince me that I will not have Bilharzia after treatment even when you tell me that I don't have Bilharzia. I will allow but I will remain worried until after sometime when nothing has changed on your body and you are very okay. Male participant, KII, fishing community.

There were concerns about adverse effects after a CHI-S including worrying about developing the disease (schistosomiasis):

… I am worried, you can tell me that you have treated me and I don't have Bilharzia yet I still have. That is where the problem is. Male participant, KII, fishing community.

There was one participant who took comfort in the fact that the information document shared with them mentioned that the planned study had been conducted elsewhere:

At first, I was worried that one can easily get problems when he is exposed but when you said that whites [not Ugandans] were also exposed … and that health workers will be there all the time to treat you when you get problems I think I can participate. Female participant, KII, fishing community.

Participants during the GDs and individual interviews mentioned the need to monitor the volunteers in the CHI-S model to avoid contact with lake water especially the fishermen who are most times on the lake. Two participants from the fishing community, during a consultative discussion, suggested having health workers tasked with visiting volunteers during the future CHI-S study. They indicated that this would be particularly important for fishermen to encourage them to avoid coming into contact with the lake water and to tell them about the dangers of coming into contact with the lake water during the CHI-S. A local leader during a key informant interview suggested involving community leaders in the CHI-S study because these are well-suited to know which volunteering fishermen are coming into contact with lake water against the CHI-S procedures.

As [local] leaders, we have contacts of fellow leaders from other fishing villages so when we get involved in the study and we know people participating in the study, we have the capacity to monitor them from all areas they go to fish. Male participant, KII, fishing community.

### Compensation for volunteering in the study

Participants supported the provision of transport refunds and compensation for time spent to come and attend to study requirements. They said that while they participate in the study, they would not generate income. One of the requirements during the CHI-S study is to avoid contact with lake water; participants reported that with compensation for income foregone at work, volunteers would participate in CHI with contentment. One participant said:

… you said that if he is a fisherman he will not be allowed to go in the lake and you will compensate him. I was particularly happy for this and I am sure a person will be in the study with one heart, not minding a lot about what his family will feed on. Male participant, GD, fishing community.

While discussing how volunteers should be compensated, the majority were non-committal because they felt there is no amount of money that can be said to be adequate. At the same time, each participant had their opinion on what would be befitting their time compensation. Whereas some would say 10,000 Uganda Shillings (about USD 2.7) per visit was adequate others felt that the figure of 120,000 Uganda Shillings (about USD 32), which had been proposed in the mock consent form would be appropriate. The argument was expressed by both men and women as below:

Money, there is no money that is enough. You cannot say that I have got all the money I want so I will not look for more money. Yes, this money is time compensation but you cannot say that it is adequate. Male participant, KII, Fishing community.

The good thing is that there is no inadequate money and there is no adequate money (laughs). What is enough for me is not enough for another person. We don't see things the same way. Female participant, KII, Fishing community.

Two participants from the university community discussed time compensation and reimbursement in specific terms:

Compensation is supposed to be 150,000 [Uganda Shillings, about USD 40] and that this is a fair compensation … since the study does not involve so many people, about thirty people, and does not take long, this money is not much money. Female participant, GD, university community.

I think that [120,000 Uganda Shillings, about USD 32] is a good one. If this money is just for transport and time compensation, I think this is okay. Male participant, KII, university community.

One participant was less concerned about compensation and was willing to participate in CHI-S with or without compensation.

We do this job [participation in CHI-S] on a voluntary basis and not that we are paid to come to the clinic. Personally, I don't buy that idea. Male participant, KII, Fishing community.

During one GD in the fishing community, some participants wondered why money for compensation had to be mentioned in the information sheets, in their view, this was a form of coercion or may lead to inducement of volunteers to take part in the study even when they are not committed to the set objectives.

Participants from both sites had mixed reactions on whether time compensation and transport reimbursement can be an undue inducement to take part in the study. There were some categories of people that were mentioned by participants as liable to being induced to take part in the study because of the money compensation:

Take an example of an idle youth … and you will definitely find these cases, if he is assured of getting 120,000 Uganda Shillings per visit, why not? Take another example of a student here [at the university] who has no job and gets involved in this study why not participate because of the money?. Female participant, KII, university community.

To limit people participating in CHI because of compensation, several participants advised the study team to clearly explain the study details to all potential volunteers so that they fully comprehended the study procedures and potential risks:

It needs to give someone who wants to participate in the study all details about the study and I know no one can volunteer with his intentions about the money if they understood the information. Information giving is important and it needs adequate time so people can know that they are in research for a reason but not for economic reasons. Male participant, KII, university community.

### Consenting process

Consent in this study was acceptable to all participants at both sites; However, some participants who attended the GDs mentioned that the consenting process that they went through (the mock) was quite long. Four of the participants in the KIIs reported that the lengthy process they went through during the consultative meeting which included the mock consent procedures affected their businesses or classes which they had to attend after the consultative session. One participant from the fishing community said:

It would be good if we spent about two or two hours and a half but not more than three hours because sometimes we have so many other things to do. Female participant, KII, Fishing community.

A student commented:

“ … I remember it reached time when we were going for evening classes before we finished and remember we started at 2 pm, and we were there for about three hours but it was not enough for us to finish”; “You could divide them into sessions. You could have a day, you have a session you go for a break and return back”. KII, university community.

Participants from the university community described the importance of consenting to participate in the study. One participant who was part of the consultative group said the consenting process allowed them to ask questions:

The consenting process was good. You took us through the process so well and you gave us chance to read all the materials and ask questions before we consented. When you are consenting an individual, you must give the information and they understand and you did this one so well and you even tested us whether we understood the study so I believe that the consent was good. Male participant, KII, university community.

Another participant suggested that information sharing, especially when it involves discussing study materials, needed to be given in different sessions to avoid tiring the participants.

It is important that next time if there is another meeting or even when you [research team] meet a new group you give them enough time so they can ask everything they want to ask because for this disease [Bilharzia] you need to ask and get answers from the health workers. Male participant, KII, Fishing community.

### Willingness to participate in CHI-S

There were mixed feelings about participation and some participants attached conditions for participation. This included the timing of the trial, especially from the university participants who reported that if the trial is to be conducted during the semester while students are studying this may not be feasible for their participation. Other participants mentioned the importance of involving their relatives in decision making before they joined the CHI-S model trial. Participants suggested that recruiting health workers as volunteers would encourage more people to join the trial:

… those people especially those of the medical profession because people know they deal with life and so if they are also part of the study as volunteers, people would get convinced. Male participant, GD, university community.

A few participants from the university and the fishing community were concerned that the age limit of 18–45 years for future CHI-S studies would lock several potential volunteers out. A participant from the university community, aged 49 years, mentioned that schistosomiasis affects all people the same way and therefore participating in CHI-S should be left open to whoever is willing regardless of their age:

Whoever is willing to volunteer because the vaccine is not going to be restricted to age. It is going to be applied to the community and mostly the old people are also the most affected by Bilharzia because they don't usually move away from the area. Their relocation to other areas is limited by their age whereas these below 45 years are very active, they will move away to new environments. Female participant, GD, university community.

A participant in the fishing community who was a farmer and village health team member with a keen interest in research decried the fact that he would not be allowed to participate in CHI-S because he is beyond the age limit.

… I am willing to participate but it is bad that I am beyond the maximum age required. I am very much interested in research and I am not scared of side effects including even death. Male participant, GD, fishing community.

Participant suggestions to enhance participation in a CHI-S model included; giving clear information to volunteers about the study procedures, having a shorter consenting process, giving appropriate compensation to cater for their lost income while in the research and developing an inclusion criteria without the age limit.

## Discussion

This paper presents a process of engaging communities in preparation for research towards a schistosomiasis vaccine trial using the controlled human infection model. This was undertaken to address the key ethical requirement of ensuring that the informed consent process is effective for all future volunteers. The process involved two phases of engaging the community; the first phase involved consulting on consenting materials and the second phase involved group sessions and key informant interviews.

### Engagement

This process of engagement was recommended by Ugandan stakeholders in preparation for CHI-S (Elliott et al., 2018). Similarly, other CHI studies have also recommended and conducted community engagements with the target communities in disease-endemic countries such as Zambia (Kunda-Ng’andu et al., 2021), Vietnam (Kestelyn et al., 2019), India (Vaz et al., 2019, 2020), Malawi (Kapumba et al., 2020), Kenya (Njue et al., 2018), and Gabon (Alabi et al., 2021). The studies showed that participants are able to understand the processes involved during a CHI. Our study found that CHI-S was generally a new concept to the participants who took part in this participatory qualitative study. This is not surprising considering that the first controlled human infection studies on the African continent were conducted in the mid-2010s (Shekalaghe et al., 2014). As far as we are aware, no controlled human infection study has been conducted in Uganda. The findings from the test of comprehension of study educational materials suggest a lot of attention is needed to ensure volunteers understand the CHI itself before they participate. This need to reflect and understand the processes was also emphasised in the consultations done in India (Vaz et al., 2019). A test of comprehension will be an important tool during the consenting process in forthcoming CHI studies, so that areas of uncertainty can be further addressed before enrolment.

At the same time, schistosomiasis was well known to the majority of respondents and the accurate knowledge demonstrated by the participants in the fishing community maybe partly due to the sensitisation (health education) conducted during past MDAs in the district (Wakiso) where the study site is located (Kabatereine et al., 2011). In addition, this study has shown that although schistosomiasis was often talked about in the community, rarely would the responsible microbes (worms) and the risk of being infected with the larval worms through the skin be discussed. This information is important to ensure that appropriate preventative strategies are emphasised in the communities. Communities are mainly focused on the disease outcome especially given the symptoms. This study conducted in preparation of a vaccine study has revealed that brochures with more details about the disease such as the transmission cycle of the parasite can enhance understanding among participants. This is a critical step in the informing process. However, as we share study brochures we need researcher presence or health education sometimes to clarify on what is not clear in study information (Musuva et al., 2014). Continued health education is an important aspects of raising awareness among research participants during the informed consent process (Kraft et al., 2019). In this study, the request for extended discussions with their social family and friends revealed the importance of giving enough time to the consenting process. This has been noted as important in the consenting process (Nishimura et al., 2013).

From our study findings, it is clear that vaccines, in general, are well known and generally acceptable in communities where common vaccines are given to children, young girls and pregnant women as part of national immunisation programmes (Turiho et al., 2017). A study in the western part of Uganda showed that the involvement of mothers enhances adhering to prompt vaccination of their children (Vonasek et al., 2016). Pregnant women who have experienced maternal vaccination are willing to receive new vaccines If they are sensitised about new vaccines (Kajungu et al., 2020). The participant willingness to take part in a vaccine trial targeting schistosomiasis has also been noted in several fishing communities in Uganda (Sanya et al., 2017). This acceptability is encouraging for efforts to develop a vaccine against schistosomiasis.

### Ethical issues

Intentional infections of healthy volunteers in any low-income, disease endemic area raise ethical issues such as risks to individual volunteers and third parties, participant selection, benefits and social value, and payments. As such, ethical considerations in safely implementing CHI studies go beyond standard international clinical trial practices such as those laid out in the Declaration of Helinski by the World Medical Association (Bambery et al., 2016).

### Risks to individual volunteers and third parties

Intentionally infecting healthy volunteers comes with risks to the individual volunteers themselves and their families or communities (the third party). Individual risks include symptoms from the intentional infection and the exposure to natural infection of volunteers enrolled in a CHI-S. These risks of CHI-S were discussed drawing from the work done in the Netherlands (Langenberg et al., 2020). The CHI-S is designed to ensure volunteers are safe by immediately treating them when infection is serologically detected before symptoms develop (Langenberg et al., 2020). In our study, participants from the fishing community mentioned that praziquantel given for the treatment of schistosomiasis had side effects. These side effects are common among the heavily infected individuals. The infection intensity in CHI-S study participants is expected to be low as few cercariae are used to infect the volunteers (Langenberg et al., 2020). Thus, treatment of CHI-S volunteers who become infected is expected to lead only to mild effects that can readily be managed by the study team. Nevertheless, it will be important for volunteers to understand, and to accept, that they will be treated with praziquantel as part of the study, and for the researchers to be sure that they understand its potential side effects.

For a CHI-S, exposure to natural infection of volunteers enrolled may encourage mixed sex infection and may lead to egg deposition and associated immunopathology. Because of the risk associated with reinfection due to contact with the lake, volunteers enrolled on a CHI-S trial have to avoid exposure through lake water contact (Elliott et al., 2018). This is not an entirely easy process as reported by the participants in the study. Discussions on this issue revealed that it may not be easy to effect especially for the participants whose livelihood and sustainability depends on the lake. For the 12-week-long CHI-S, avoidance of exposure with schistosomes would need to be emphasised when potential volunteers are screened and/or enrolled. This impacts on the freedom of the volunteer and it may be an ethical dilemma since it requires a restrain on part of the volunteer to avoid their income-generating activities such as fishing. Engaging the community in this study revealed several suggestions by the participants including provision of better infrastructure such as increasing tapped water in the studied community. This emphasis of study procedures is important during the consent process because people are used to their “usual” daily activities such as fishing. A pragmatic alternative may be to involve fishing community members whose livelihood does not absolutely require lake contact.

Additionally, treating all volunteers immediately infection is detected by serological means during the CHI-S, and treating all volunteers at the end of the study prevents the spread of the *S. mansoni* strain used in the CHI-S to the wider community. This fulfils an ethical requirement to protect third parties (the community) from harm (Binik, 2020). Relevant third parties of CHI studies include close family and friends and communities of CHI volunteers. The study participants were allowed a week to consult with their family members and this study showed that potential volunteers would want to seek advice from their close trusted social networks such as family members before they commit to a decision to take part in the research. Family involvement in decision making for medical interventions such as treatment and immunisation of study participants has been expressed in a past study on perceptions on vaccine safety among caretakers of young children (Braka et al., 2012). Family involvement is a common cultural value in communities with relational interdependence and social hierarchy (Alden et al., 2018). This underlines the importance of understanding and respecting the cultural and societal norms and cohesion of the target community by researchers who propose research in a given target community.

### Participant selection

Participant selection for CHI gives prominence to the ethical need for equity in the process (Bambery et al., 2016) and exploitation of vulnerable groups (Rose, 2018). Purposeful participant inclusion and exclusion enables that those selected are able to understand the nature of the CHI-S (Rose, 2018) and covers a representative group of people (Vaz et al., 2020). The former allows particpants to raise their views and concerns as seen elsewhere (Hodgson et al., 2015).

### Benefits and social value

For studies such as CHI where there is no direct benefit to the individual volunteer, social value of the research takes prominence (Rid & Shah, 2017). The participants in this study agreed that their involvement would contribute to new knowledge to develop a vaccine to free their families and community from schistosomiasis. Hoogerwerf et al. have shown that the desire to contribute to science, and medical research, as well as financial compensation, were motivating factors among schistosomiasis naïve volunteers of controlled human infection studies over 80% of whom were proud to participate (Hoogerwerf et al., 2020). Similarly, altruistic motivation was also observed in controlled human malaria infection studies (Kraft et al., 2019).

### Payments

Current discussions acknowledge that payments in CHI studies need to be fair, adequate and of the right type (either compensation, reimbursement or incentive) (Lynch et al., 2021). The fair and adequate amount of payment is difficult to determine even with input from the stakeholders. This may partly be due to participants analysing the balance between the risks/burdens and the benefits of participation. In our study, most participants were non-committal as to the actual amount in Uganda Shillings that would be appropriate for compensation if they were to participate in the CHI study. Similarly, stakeholders for a Malawian CHI were noncommittal on compensation rates (Kapumba et al., 2020). This shows the difficulty in allocating a monetary value to time and effort in the informal sector. The amount of money that appeared in the information document for study participants as compensation for time bothered some participants as it was seen as a form of inducement to take part in the study. Indeed undue inducement has been cited as a major ethical challenge for CHI studies in endemic settings (Selgelid & Jamrozik, 2018). Monetary compensation was found to strongly motivate participation in the controlled human malaria infection studies (Kraft et al., 2019; Njue et al., 2018)The amount for payment that is acceptable widely varies. In public consultations for CHI for COVID-19, weekly payment was considered adequate if higher than weekly wages but inadequate when the risks of complications were considered (Gbesemete et al., 2020). The financial situations of volunteers such as the low socioeconomic status of people in target endemic areas such as fishing communities (for CHI-S) may lead to undue inducement, making it difficult to decide on the most appropriate payment (Selgelid & Jamrozik, 2018) and payment type (Ndebele & Hyder, 2021). In our study, a consensus was not reached on the level of compensation. This debate on inducement requires further research to underscore the importance and implication of compensation to volunteers during CHI studies in endemic communities. It is important to discuss payments in preparatory studies (Jamrozik & Selgelid, 2020b).

There were limitations in this study**.** The number of the participants was small and so results may not be fully generalisable, but the shared insights about the educational materials, informed consent and volunteer perception of a CHI-S model in two different communities will be useful to researchers planning on conducting a CHI-S. The inclusion criteria for age of 18–50 years for the current study was difficult to observe in both communities and a few older participants were included. The suggestion to emphasise exposure to schistosomiasis in water may outweigh the need to have an age limit for participating respondents. In addition, older people are more likely to have other (possibly undiagnosed) health problems than their younger counterparts. In the current study, it was possible that the selection of participants by the peer leaders in the communities could have influenced their willingness to participate, despite the private consenting process, and could possibly bias the findings. Other studies in endemic setting have had careful consideration of participant selection using convenient sampling (Njue et al., 2018). Controlled human infection studies have a long history that includes well-documented incidences that resulted into injury and sometimes death (Schaefer 2004, Hope and McMillan 2004). A limitation of our study is that we did not include information on historical examples of serious and fatal challenge studies in the information provided to participants. Consideration will be given to this in future. However, of note, the probability of such an event is low for schistosomiasis where severe pathology and fatal outcomes are generally the results of repeated exposure and prolonged, untreated infection.

There are several strengths of this study. To the best of our knowledge, this study was the first of its kind in Uganda to engage with local communities to discuss controlled human infections studies and how these can be used for vaccine trials. Secondly, the study was conducted following recommendations of stakeholders which was the first phase of engaging with the community (Elliott et al., 2018). When done early in the establishment of a CHI model, engaging stakeholders adapt the study design and the participant selection, consent and payment. In the present study, two target communities were able to discuss and contribute to developing informed consent procedures and educational materials. The input of the communities in the educational material development and adaptation process was very important to contextualise the materials and procedures. This makes consultation of target community on study processes and procedures vital to develop community-specific research protocols (Dickert & Sugarman, 2005). This study did not only collect data on perceptions, but it also combined learning and discussing prepared study materials and the researchers allowed for the integration of suggestions made by the participants and input from the consultative meetings to be adapted in planned CHI-S studies.

## Conclusion

The implementation of a CHI-S in Uganda is planned, with the aim of fast-track testing schistosomiasis vaccine candidates in a schistosomiasis endemic population. Communities are willing to take part in a CHI-S study. However, there is a need to give enough time for the informed consent process to share and ensure understanding of study procedures, risks, and benefits. Volunteer time compensation in a CHI study remains a problematic issue, to minimise the risk of undue inducement among the potential volunteers. Further community engagement to assess the dynamics of participating in a CHI study will be crucial during and after the CHI-S is implemented for a vaccine trial in the endemic communities.

## Supplementary Material

Supplemental Material

Supplemental Material

Supplemental Material

Supplemental Material

Supplemental Material

Supplemental Material

Supplemental Material

## Data Availability

The authors confirm that the data supporting the findings of this study are available within the article and its supplementary materials.
