# Supplementary material for: Ethical and practical considerations arising from community consultation on implementing controlled human infection studies using Schistosoma mansoni in Uganda
Source: Glob Bioeth. 2022 Jul 4;33(1):78–102. doi: 10.1080/11287462.2022.2091503 (PMC9258062; doi:10.1080/11287462.2022.2091503)
Supplement: Supplemental Material [file RGBE_A_2091503_SM9958.doc]

**Supporting information**

**S3. Guide for group discussions**

| **Developing informed consent procedures for Controlled Human Infection studies using *Schistosoma mansoni* in Ugandan populations** | |
| --- | --- |
| **CHI-S Study** | |
| **GUIDE FOR GROUP DISCUSSIONS** | |
| 1 | Assess how well participants understood the CHI-S model, its purpose, its risks to volunteers, its potential societal benefits? |
|  |  |
| 2 | Assess the attitudes to the risks involved in CHI-S participation |
|  |  |
| 3 | Assess how educational materials presented, and consent process, could be improved |
|  |  |
| 4 | What attitude the participants now have to CHI-S – whether they think it should be implemented in Uganda, whether they would participate themselves and whether they would support their friends and family to take part in such a study? |
|  |  |
| 5 | Assess the implications of participation for time off work, studies and daily responsibilities for clinic visits, and the feasibility of this |
|  |  |
| 6 | Assess the likely costs, or financial losses, that would be incurred |
|  |  |
| 7 | Assess the feasibility of avoiding contact with contaminated water during the 12-week CHI-S studies |
|  |  |
| 8 | Assess the attitudes to compensation for participation in CHI-S and expectations as to what this would or should be |
|  |  |
