# Supplementary material for: Ethical and practical considerations arising from community consultation on implementing controlled human infection studies using Schistosoma mansoni in Uganda
Source: Glob Bioeth. 2022 Jul 4;33(1):78–102. doi: 10.1080/11287462.2022.2091503 (PMC9258062; doi:10.1080/11287462.2022.2091503)
Supplement: Supplemental Material [file RGBE_A_2091503_SM9949.doc]

**Supporting information**

**S2. Guide for key info**rmant interviews

| **Developing informed consent procedures for Controlled Human Infection studies using *Schistosoma mansoni* in Ugandan populations** | |
| --- | --- |
| **CHI-S Study** | |
| **GUIDE FOR KEY INFORMANT INTERVIEWS** | |
| **Topics to guide interview** | |
| 1 | Background information |
|  | *Ask about the respondent’s background: age, gender, level of education, occupation, tribe, marital status, other household members in the family, how long the respondent has lived in the fishing community/the university community, housing, method of disposal of stool, any occupant in the household who is ill/sick* |
| 2 | Assessing awareness of existence, causes, transmission, health problems and control of schistosomiasis |
|  | *Assess the respondent’s knowledge (ask about health problems in community, what is most important, if Bilharzia is not mentioned ask whether it exists and their experience with it).*  *Ask respondent to compare schistosomiasis to other health issues in terms of its effect in the community)*   - Tell me about your experience living in the community where Bilharzia is a challenge (follow this appropriately if participant has already mentioned it, ask for any experience with a member of family who suffered from Bilharzia, what did it mean to family in terms of cost, day-to-day activities of the individual and other members in family) - Probe for causes, transmission and risks for contracting schistosomiasis - Probe for symptoms and signs of schistosomiasis, and how it can be prevented - Probe about barriers to control schistosomiasis in the community |
| 3 | Assessing attitudes towards the development of vaccines to control schistosomiasis |
|  | - What do you know about vaccines and how they work? |
| 4 | How well participants have understood the CHI-S model, its purpose, its risks to volunteers, its potential societal benefits |
|  | - What do you know about the CHI-S model? In your view why do you think there is a suggestion for this model, what may be the risk to volunteers who take part in research using this model - How do you think the society/community as a whole may benefit if this kind of research is conducted in the community? |
| 5 | Attitudes to the risks involved in CHI-S participation |
|  | - What would be the reasons for taking part or refusing to take part in this kind of research (*probe for pros and cons*) - Given that people need to get to the lake for various reasons including fishing, washing clothes, collecting water for domestic use; Do you think prolonged stay away from water (12 weeks) is possible? (*Explain this)* |
| 6 | How educational materials presented, and consent process, could be improved |
|  | - What did you like the most in the educational materials presented? - What did you not like in the educational materials presented? |
| 8 | Assess the implications of participation for time off work, studies and daily responsibilities for clinic visits, and the feasibility of this |
|  | - Do you think you can take off time from work, studies and daily responsibilities for these sessions? |
| 9 | Assess the likely costs, or financial losses, that would be incurred |
|  | - If you were to participate in this study, approximately how much money would you lose per day? If you were asked to be away from work for 12 would this be feasible, why?   You may probe for how much money they would lose if they were away, ask them to describe this per day (*this is sensitive and needs not be rushed*) |
| 10 | Assess the feasibility of avoiding contact with contaminated water during the 12-week CHI-S studies |
|  | - The study may require one to avoid direct contact with lake water for at least 12 weeks, do you think people may accept this/ what about you? |
| 11 | Assess the attitudes to compensation for participation in CHI-S and expectations as to what this would or should be |
|  | - Let us talk about compensation; do you think it is important to compensate people for their time during research such as the CHI-S? - What in your view would be the amount that one should be compensated for their time? (*wait for responses before you add the following question*). - Currently, some research may compensate between 20,000-25,000 UGX in your view is this adequate for your time to participate in CHI-S? |
| 12 | Thank you very much for your time |
