# Supplementary material for: Ethical and practical considerations arising from community consultation on implementing controlled human infection studies using Schistosoma mansoni in Uganda
Source: Glob Bioeth. 2022 Jul 4;33(1):78–102. doi: 10.1080/11287462.2022.2091503 (PMC9258062; doi:10.1080/11287462.2022.2091503)
Supplement: Supplemental Material [file RGBE_A_2091503_SM9838.doc]

**Supporting information**

**S1. Testing of comprehension questions**

**CHI-S Study Form**

**Testing Comprehension**

By testing comprehension, we want to determine whether participants correctly understand the aspects of the study. Please circle ONE correct answer to each of the following questions.

1. We get Bilharzia from
   1. eating uncooked food
   2. going to the lake water
   3. walking barefooted
   4. drinking un boiled water
2. Bilharzia can be stopped by minimizing contact with infested water
   1. Yes
   2. No
3. A vaccine could prevent Bilharzia
   1. True
   2. False
4. In a controlled human infection study,
   1. the study is explained to volunteers to understand the purpose, procedures, benefits and potential problems
   2. volunteers are given larvae on the skin that will not produce any eggs
   3. volunteers are monitored weekly for signs of infection
   4. all the above
5. The first thing to do in a controlled human infection study is to determine the number of larvae to safely use on volunteers
   1. True
   2. False
6. Testing a vaccine using controlled human infection involves
   1. giving healthy volunteers with a vaccine being developed
   2. infecting all healthy volunteers with a germ
   3. monitoring all infected volunteers for signs of infection
   4. treating all volunteers at the end of the study
   5. all the above
7. Volunteers come into the clinic every week after infection for
   1. a general health check-up
   2. blood, urine and faeces tests
   3. all the above
8. A controlled human Bilharzia infection may cause symptoms in CHI-S volunteers
   1. Yes
   2. No
9. Before a volunteer can participate in the study he/she needs to:
   1. fully understand the purpose, procedures, benefits and potential problems
   2. consult with family and friends
   3. be very healthy
   4. consent to volunteer in the study
   5. all the above
10. Do volunteers personally benefit from participating in CHI?
    1. Yes
    2. No
