# Supplementary material for: Ethical and practical considerations arising from community consultation on implementing controlled human infection studies using Schistosoma mansoni in Uganda
Source: Glob Bioeth. 2022 Jul 4;33(1):78–102. doi: 10.1080/11287462.2022.2091503 (PMC9258062; doi:10.1080/11287462.2022.2091503)
Supplement: Supplemental Material [file RGBE_A_2091503_SM9832.pptx]

## Slide 1
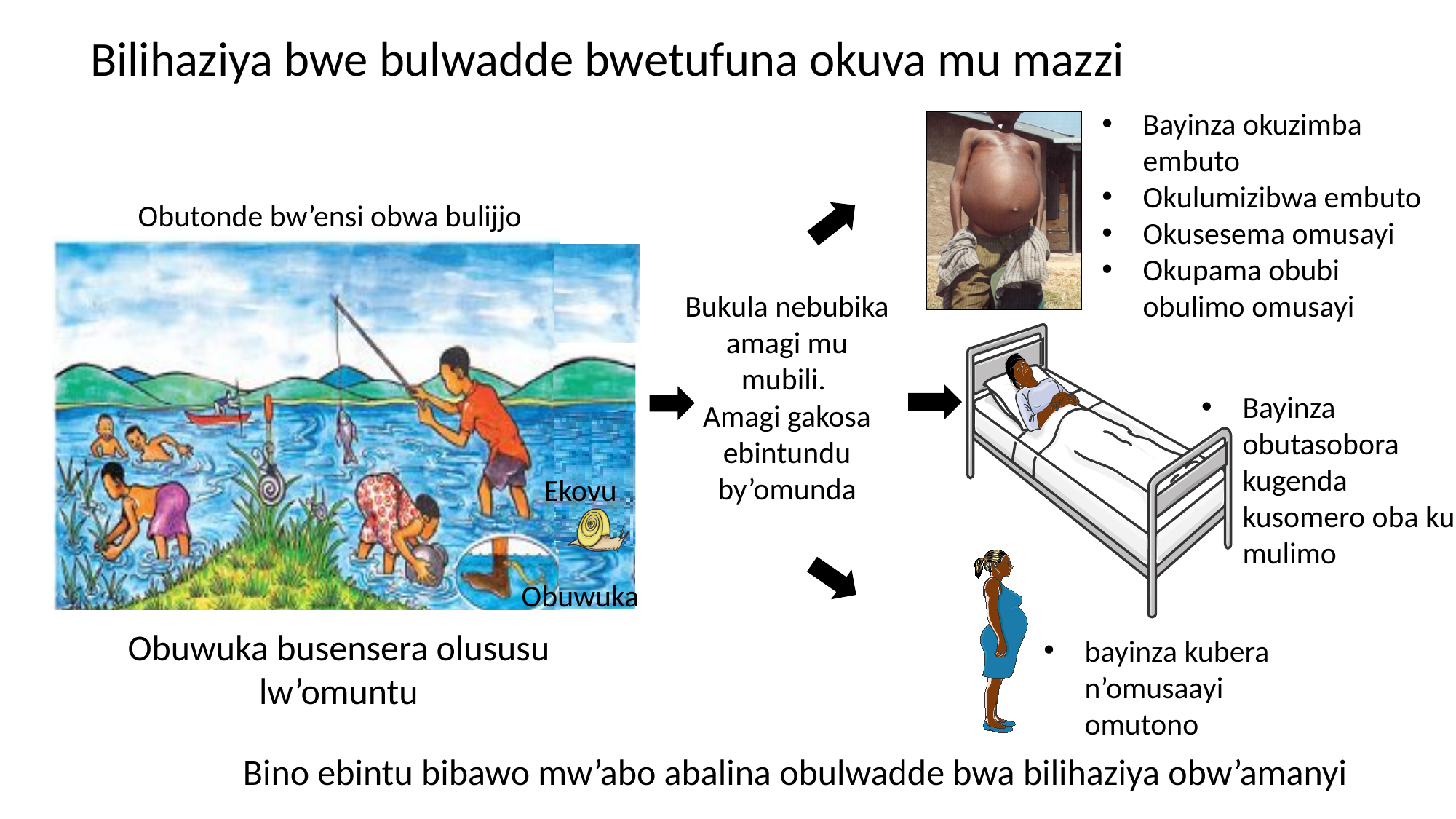

Bilihaziya bwe bulwadde bwetufuna okuva mu mazzi
Bayinza okuzimba embuto
Okulumizibwa embuto
Okusesema omusayi
Okupama obubi obulimo omusayi
Obutonde bw’ensi obwa bulijjo
Bukula nebubika amagi mu mubili.
Amagi gakosa ebintundu by’omunda
Bayinza obutasobora kugenda kusomero oba ku mulimo
Ekovu
Obuwuka
Obuwuka busensera olususu lw’omuntu
bayinza kubera n’omusaayi omutono
Bino ebintu bibawo mw’abo abalina obulwadde bwa bilihaziya obw’amanyi

## Slide 2
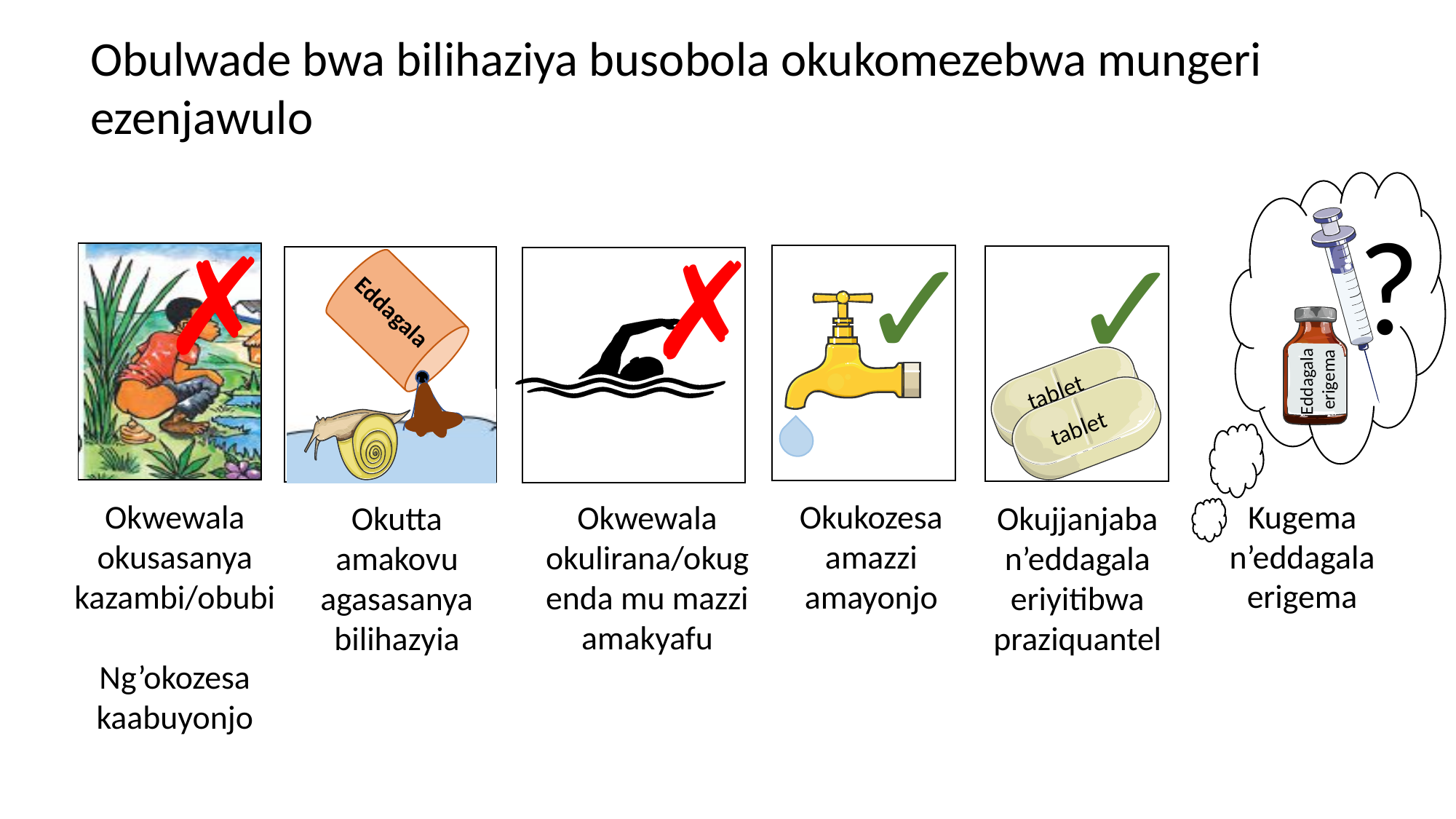

Obulwade bwa bilihaziya busobola okukomezebwa mungeri ezenjawulo
?
✗
✓
✓
✗
Eddagala
Eddagala
 erigema
tablet
tablet
Kugema n’eddagala erigema
Okukozesa amazzi amayonjo
Okwewala okusasanya kazambi/obubi
Ng’okozesa kaabuyonjo
Okwewala okulirana/okugenda mu mazzi amakyafu
Okutta amakovu agasasanya bilihazyia
Okujjanjaba n’eddagala eriyitibwa praziquantel

## Slide 3
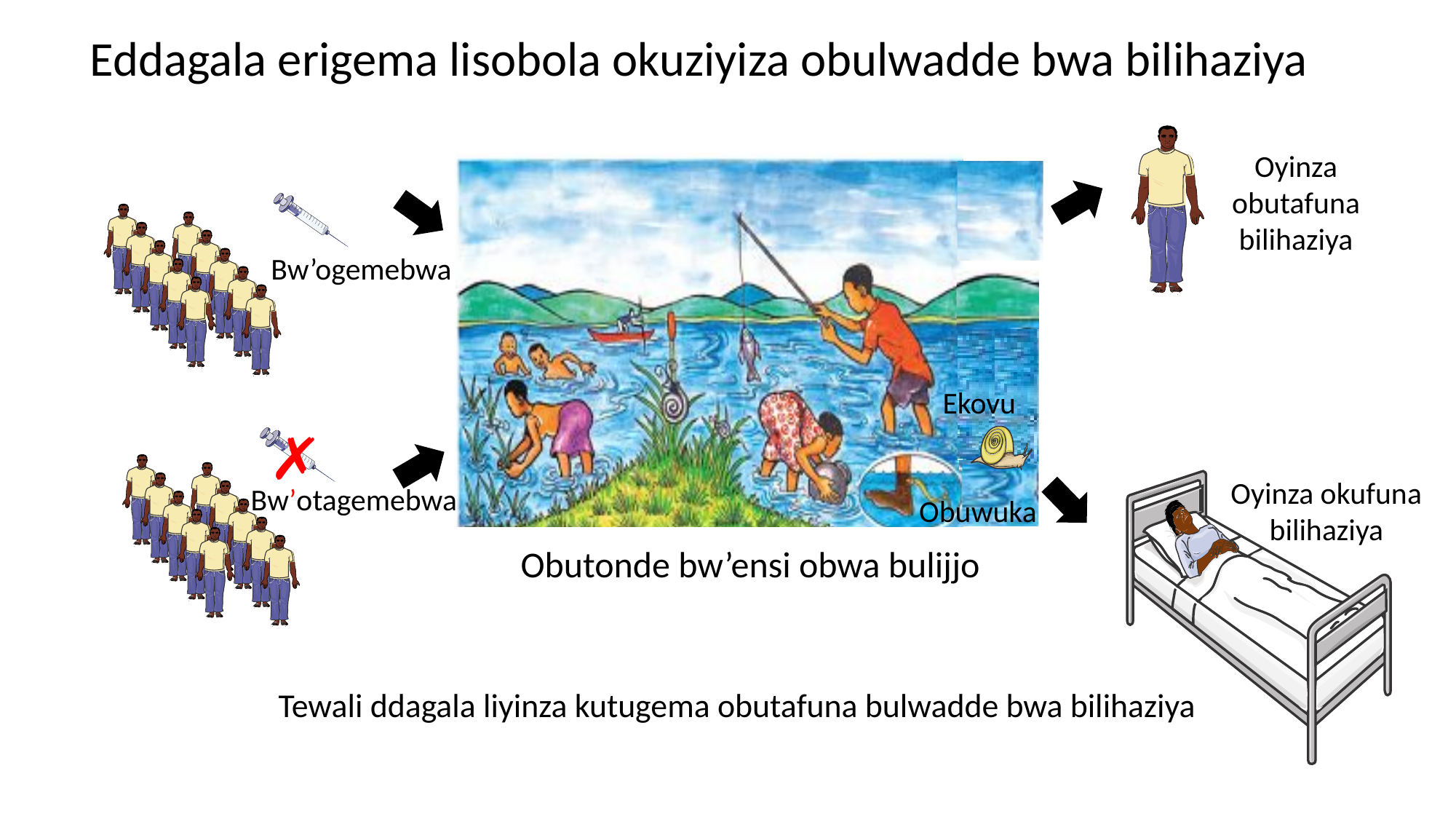

Eddagala erigema lisobola okuziyiza obulwadde bwa bilihaziya
Oyinza obutafuna bilihaziya
Bw’ogemebwa
Ekovu
✗
Oyinza okufuna bilihaziya
Bw’otagemebwa
Obuwuka
Obutonde bw’ensi obwa bulijjo
Tewali ddagala liyinza kutugema obutafuna bulwadde bwa bilihaziya

## Slide 4
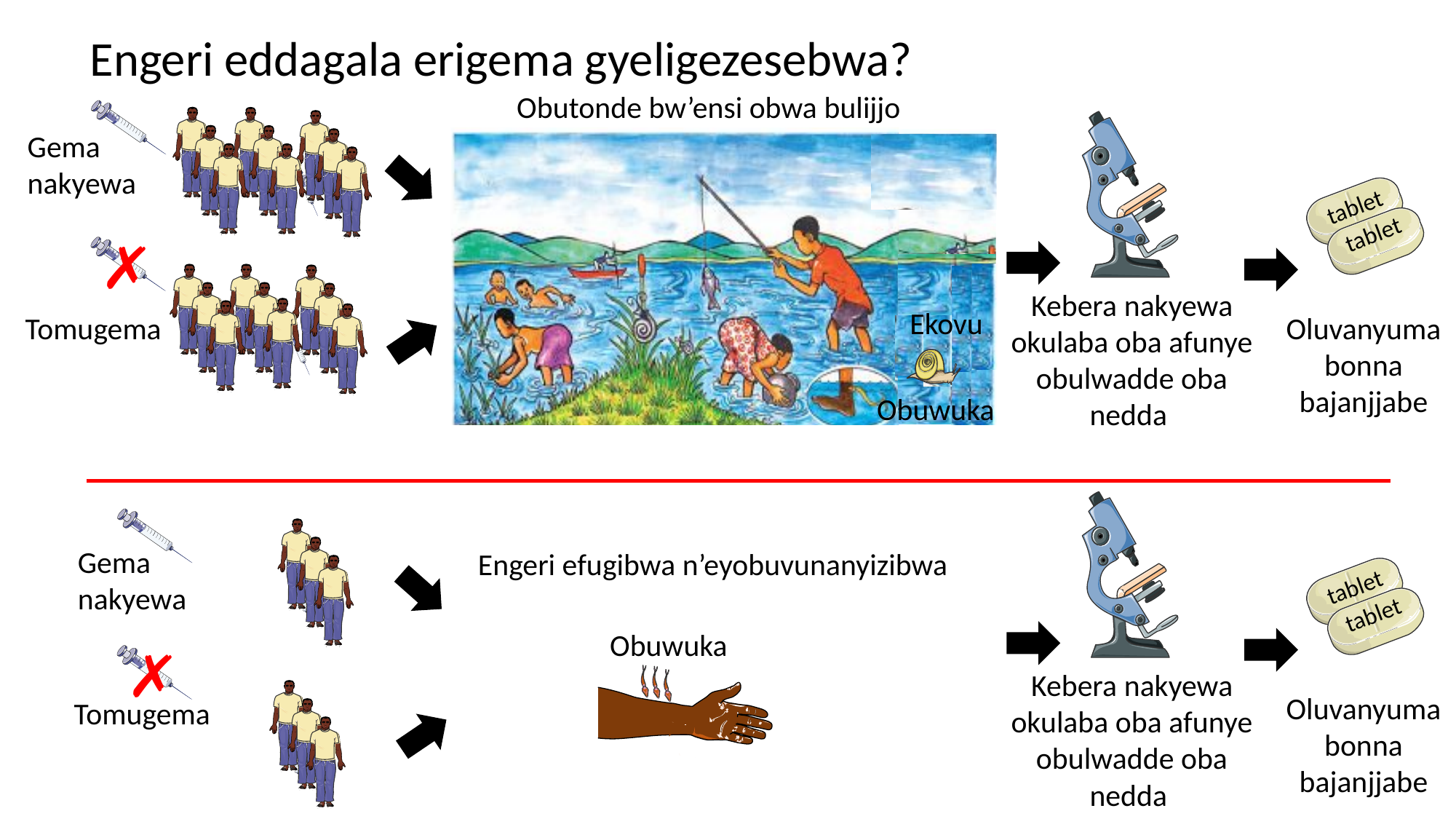

Engeri eddagala erigema gyeligezesebwa?
Obutonde bw’ensi obwa bulijjo
Gema nakyewa
tablet
tablet
✗
Kebera nakyewa okulaba oba afunye obulwadde oba nedda
Ekovu
Tomugema
Oluvanyuma bonna bajanjjabe
Obuwuka
Gema nakyewa
Engeri efugibwa n’eyobuvunanyizibwa
tablet
tablet
Obuwuka
✗
Kebera nakyewa okulaba oba afunye obulwadde oba nedda
Oluvanyuma bonna bajanjjabe
Tomugema

## Slide 5
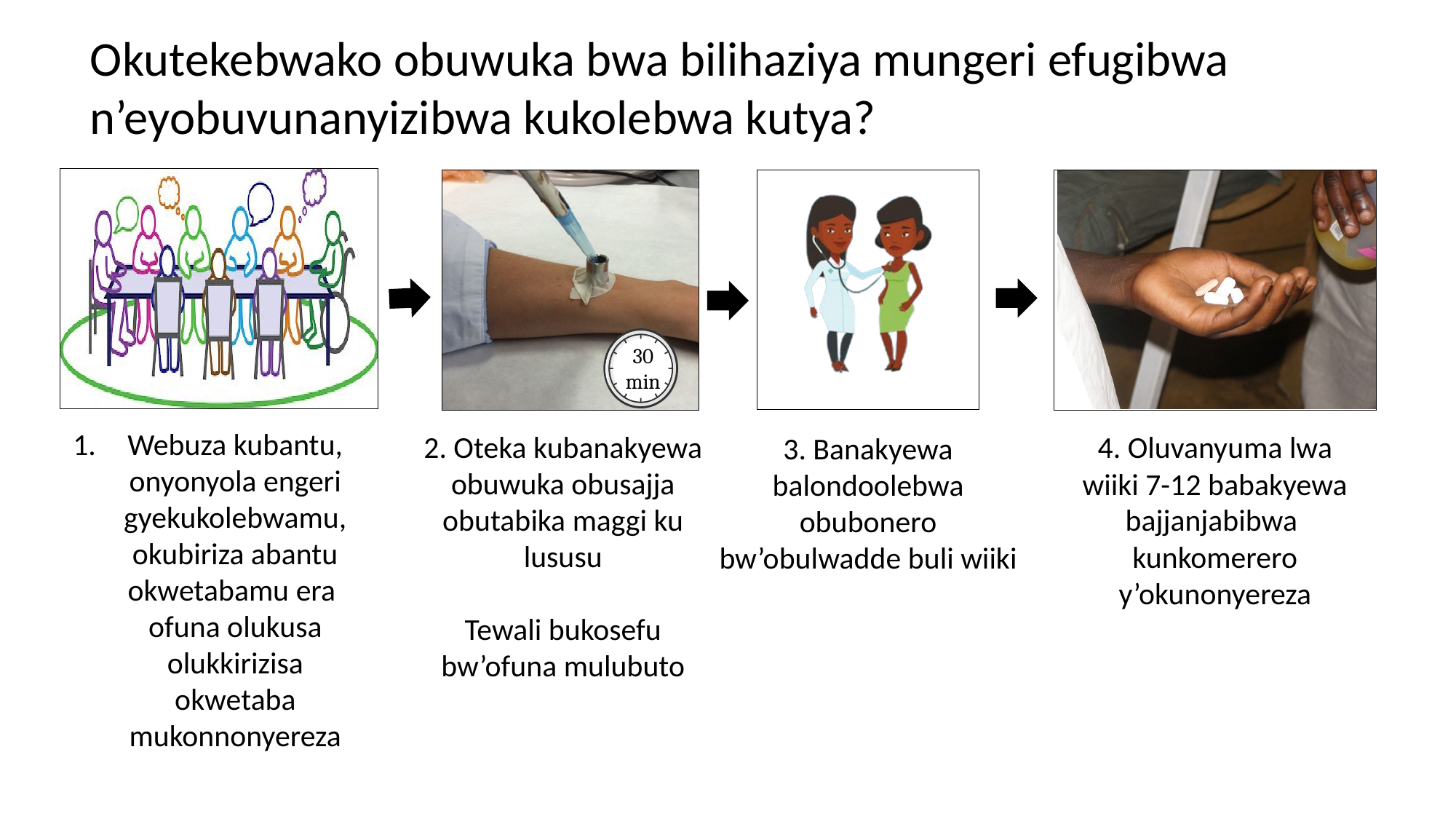

Okutekebwako obuwuka bwa bilihaziya mungeri efugibwa n’eyobuvunanyizibwa kukolebwa kutya?
30
min
Webuza kubantu, onyonyola engeri gyekukolebwamu,okubiriza abantu okwetabamu era ofuna olukusa olukkirizisa okwetaba mukonnonyereza
2. Oteka kubanakyewa obuwuka obusajja obutabika maggi ku lususu
Tewali bukosefu bw’ofuna mulubuto
4. Oluvanyuma lwa wiiki 7-12 babakyewa bajjanjabibwa kunkomerero y’okunonyereza
3. Banakyewa balondoolebwa obubonero bw’obulwadde buli wiiki

## Slide 6
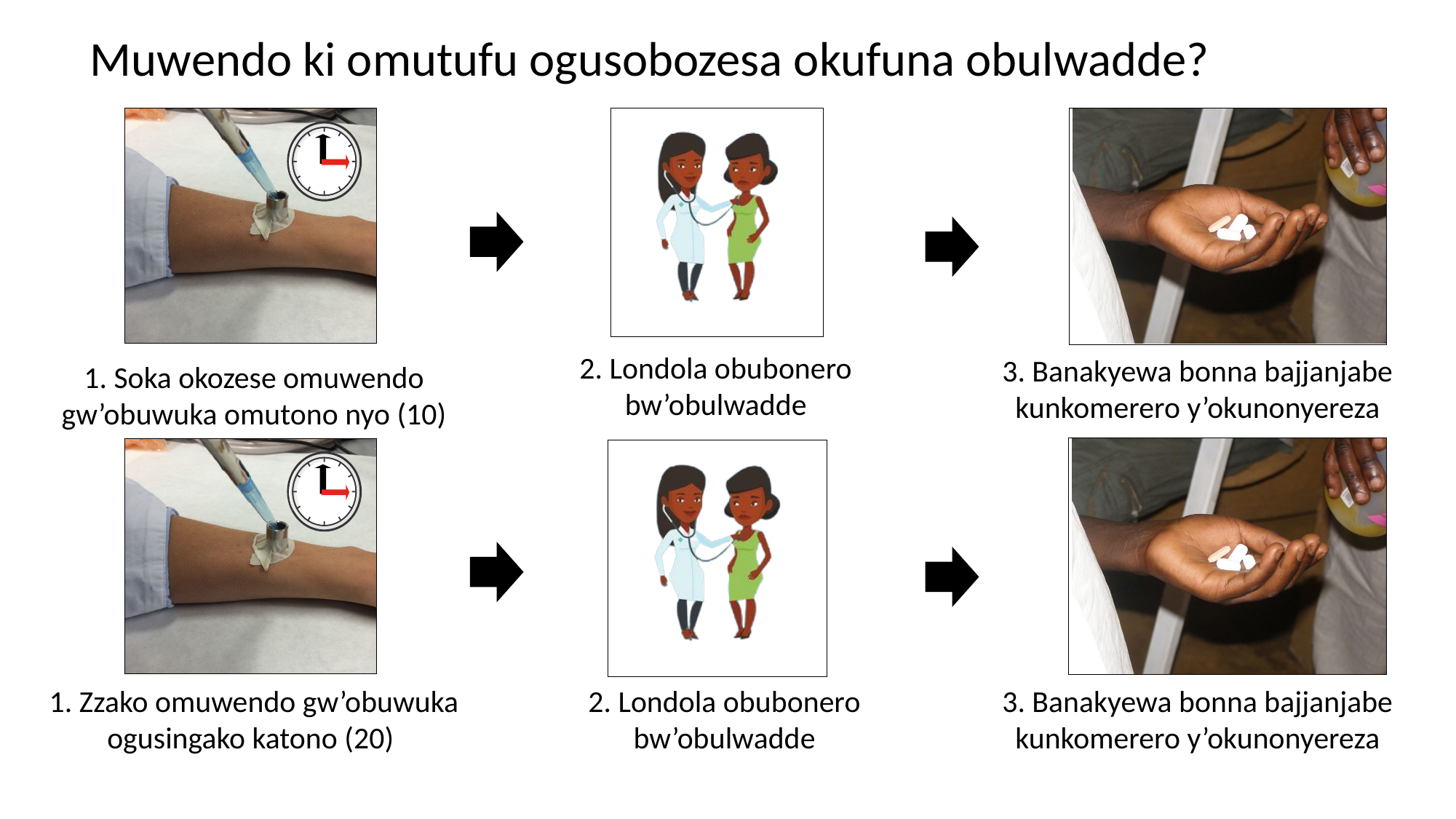

Muwendo ki omutufu ogusobozesa okufuna obulwadde?
2. Londola obubonero bw’obulwadde
3. Banakyewa bonna bajjanjabe kunkomerero y’okunonyereza
1. Soka okozese omuwendo gw’obuwuka omutono nyo (10)
1. Zzako omuwendo gw’obuwuka ogusingako katono (20)
2. Londola obubonero bw’obulwadde
3. Banakyewa bonna bajjanjabe kunkomerero y’okunonyereza

## Slide 7
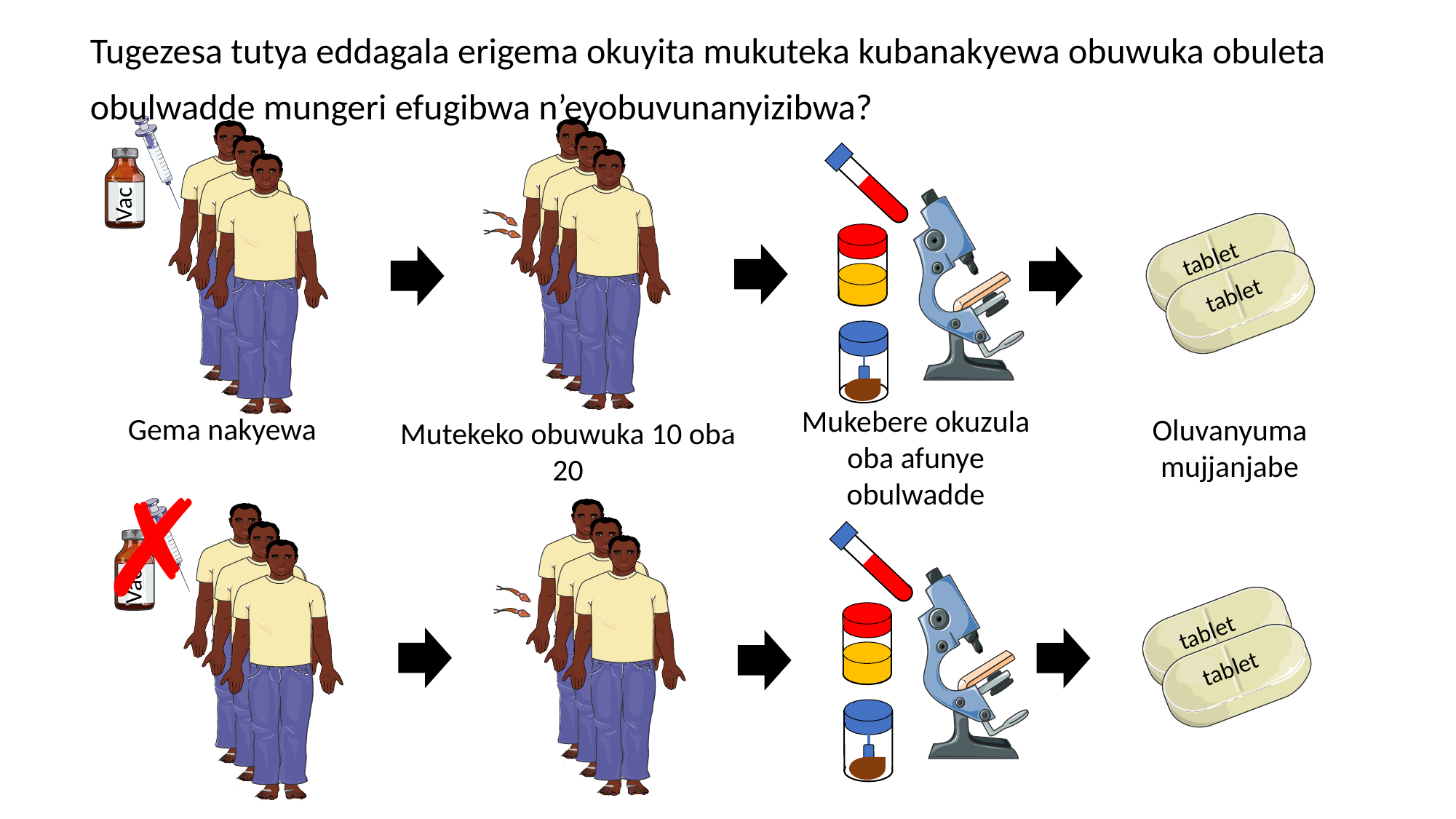

Tugezesa tutya eddagala erigema okuyita mukuteka kubanakyewa obuwuka obuleta obulwadde mungeri efugibwa n’eyobuvunanyizibwa?
Vac
tablet
tablet
Mutekeko obuwuka 10 oba 20
Mukebere okuzula oba afunye obulwadde
Gema nakyewa
Oluvanyuma mujjanjabe
✗
Vac
tablet
tablet

## Slide 8
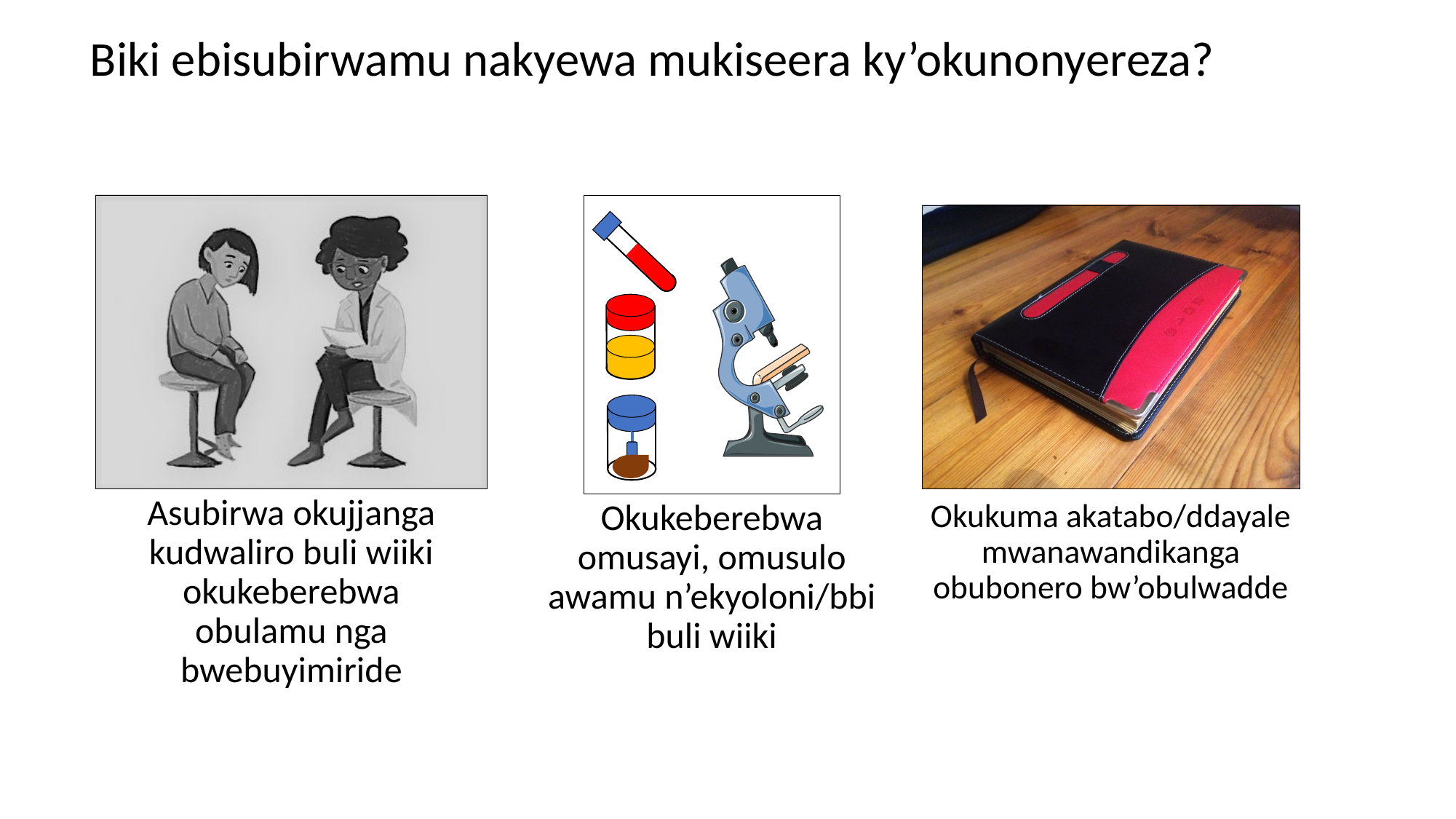

Biki ebisubirwamu nakyewa mukiseera ky’okunonyereza?
Asubirwa okujjanga kudwaliro buli wiiki okukeberebwa obulamu nga bwebuyimiride
Okukeberebwa omusayi, omusulo awamu n’ekyoloni/bbi buli wiiki
Okukuma akatabo/ddayale mwanawandikanga obubonero bw’obulwadde

## Slide 9
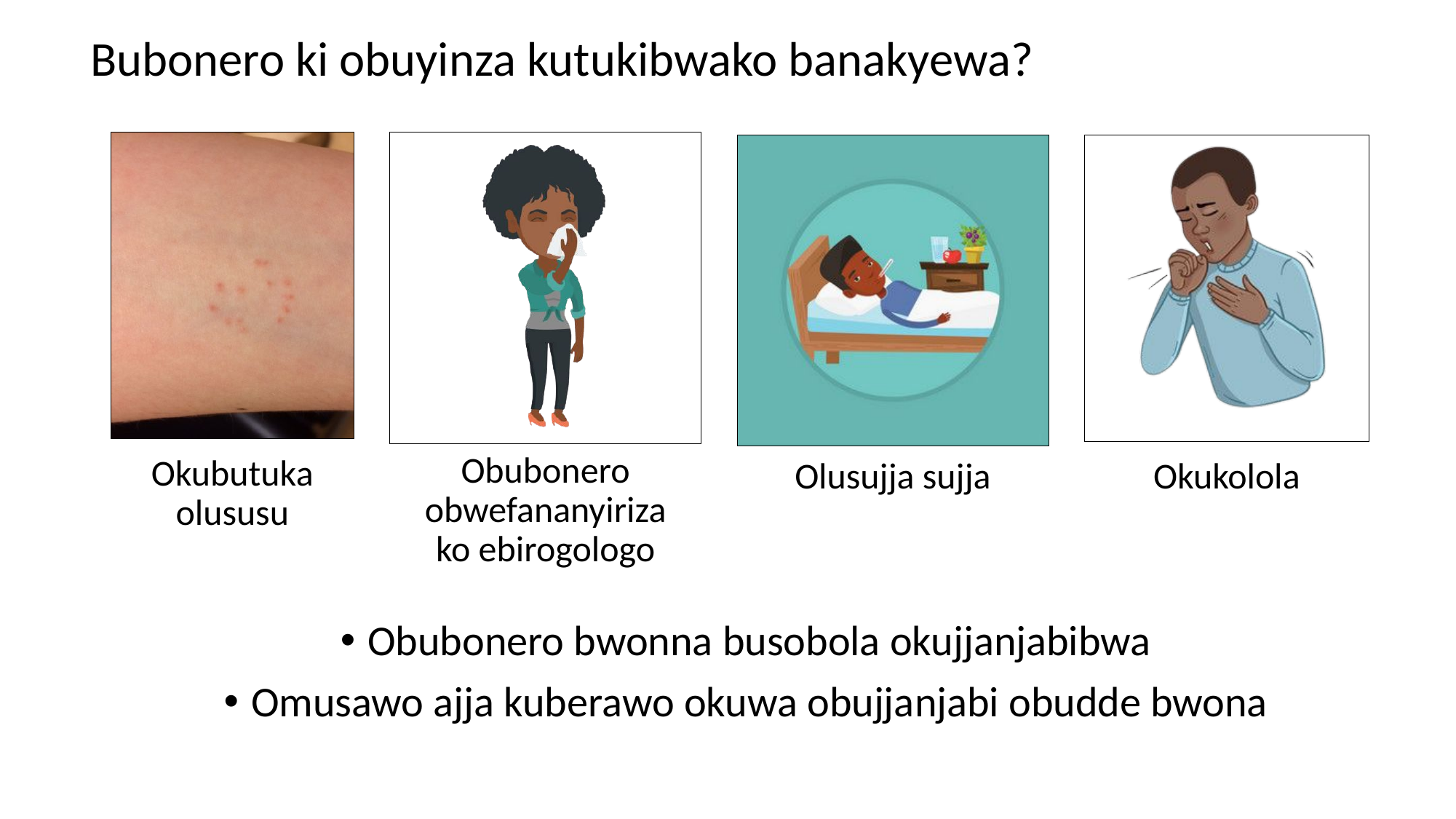

Bubonero ki obuyinza kutukibwako banakyewa?
Obubonero obwefananyirizako ebirogologo
Olusujja sujja
Okukolola
Okubutuka olususu
Obubonero bwonna busobola okujjanjabibwa
Omusawo ajja kuberawo okuwa obujjanjabi obudde bwona

## Slide 10
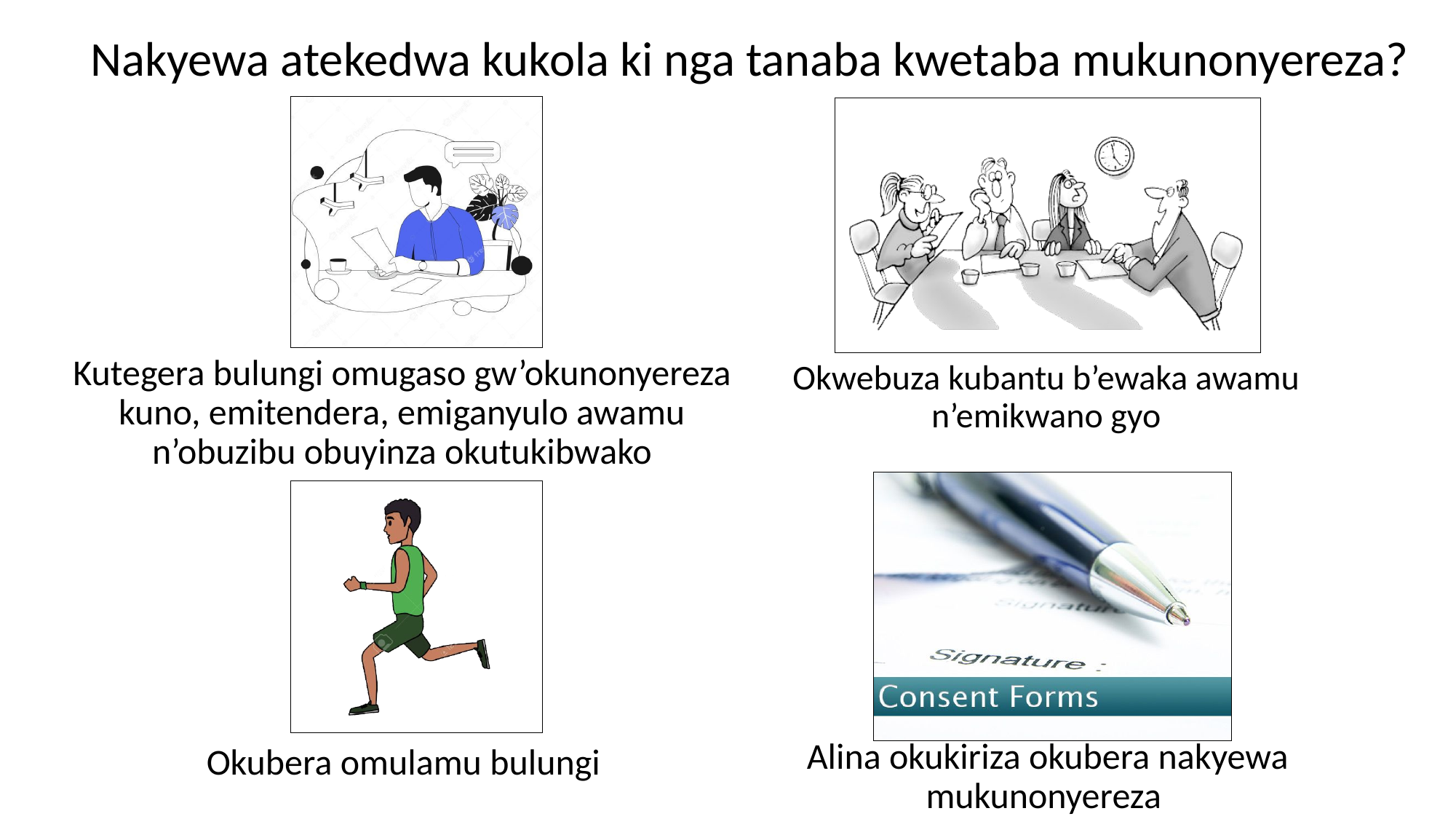

Nakyewa atekedwa kukola ki nga tanaba kwetaba mukunonyereza?
Kutegera bulungi omugaso gw’okunonyereza kuno, emitendera, emiganyulo awamu n’obuzibu obuyinza okutukibwako
Okwebuza kubantu b’ewaka awamu n’emikwano gyo
Alina okukiriza okubera nakyewa mukunonyereza
Okubera omulamu bulungi

## Slide 11
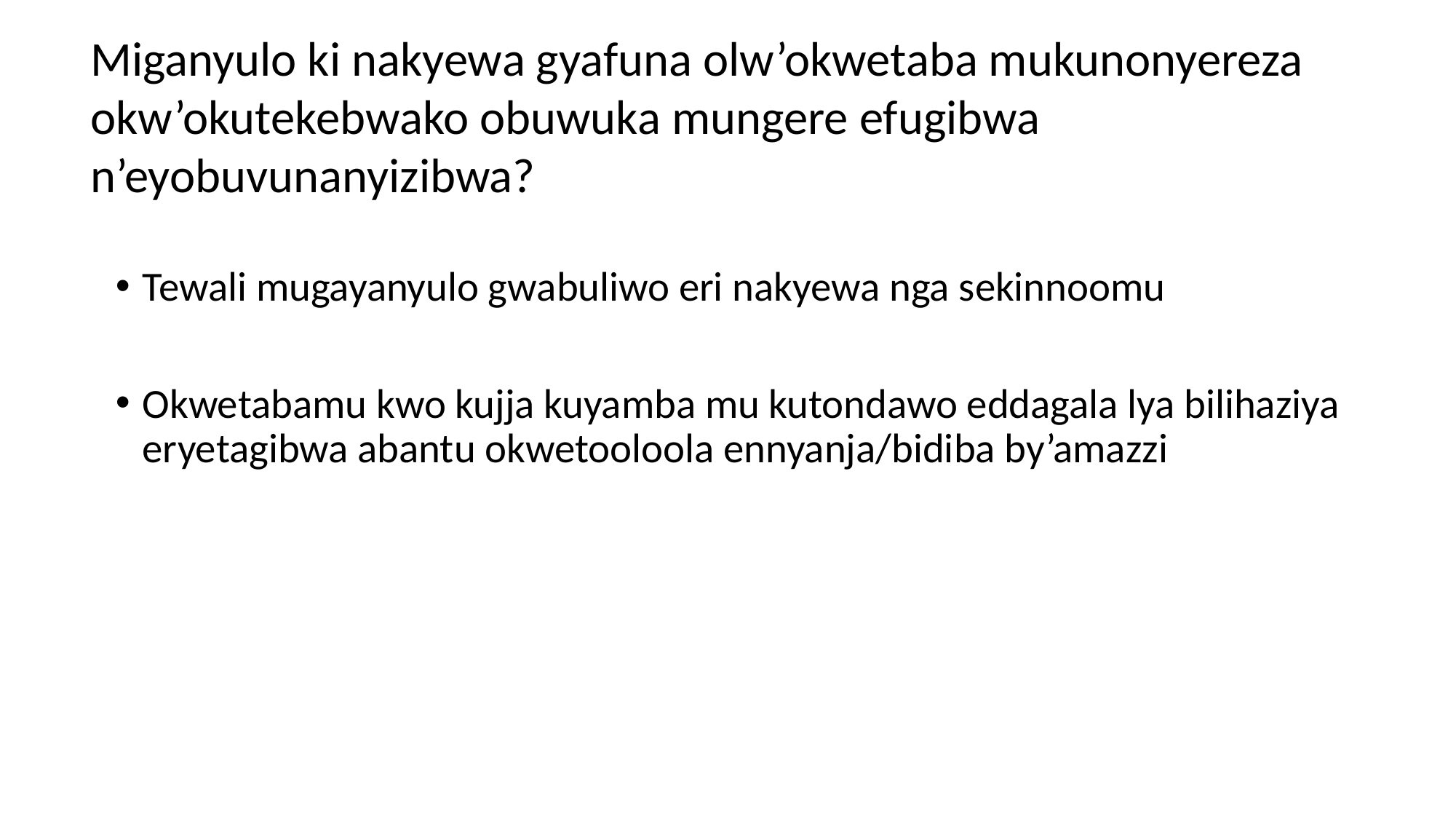

Miganyulo ki nakyewa gyafuna olw’okwetaba mukunonyereza okw’okutekebwako obuwuka mungere efugibwa n’eyobuvunanyizibwa?
Tewali mugayanyulo gwabuliwo eri nakyewa nga sekinnoomu
Okwetabamu kwo kujja kuyamba mu kutondawo eddagala lya bilihaziya eryetagibwa abantu okwetooloola ennyanja/bidiba by’amazzi
