# Supplementary material for: Ethical and practical considerations arising from community consultation on implementing controlled human infection studies using Schistosoma mansoni in Uganda
Source: Glob Bioeth. 2022 Jul 4;33(1):78–102. doi: 10.1080/11287462.2022.2091503 (PMC9258062; doi:10.1080/11287462.2022.2091503)
Supplement: Supplemental Material [file RGBE_A_2091503_SM9821.pptx]

## Slide 1
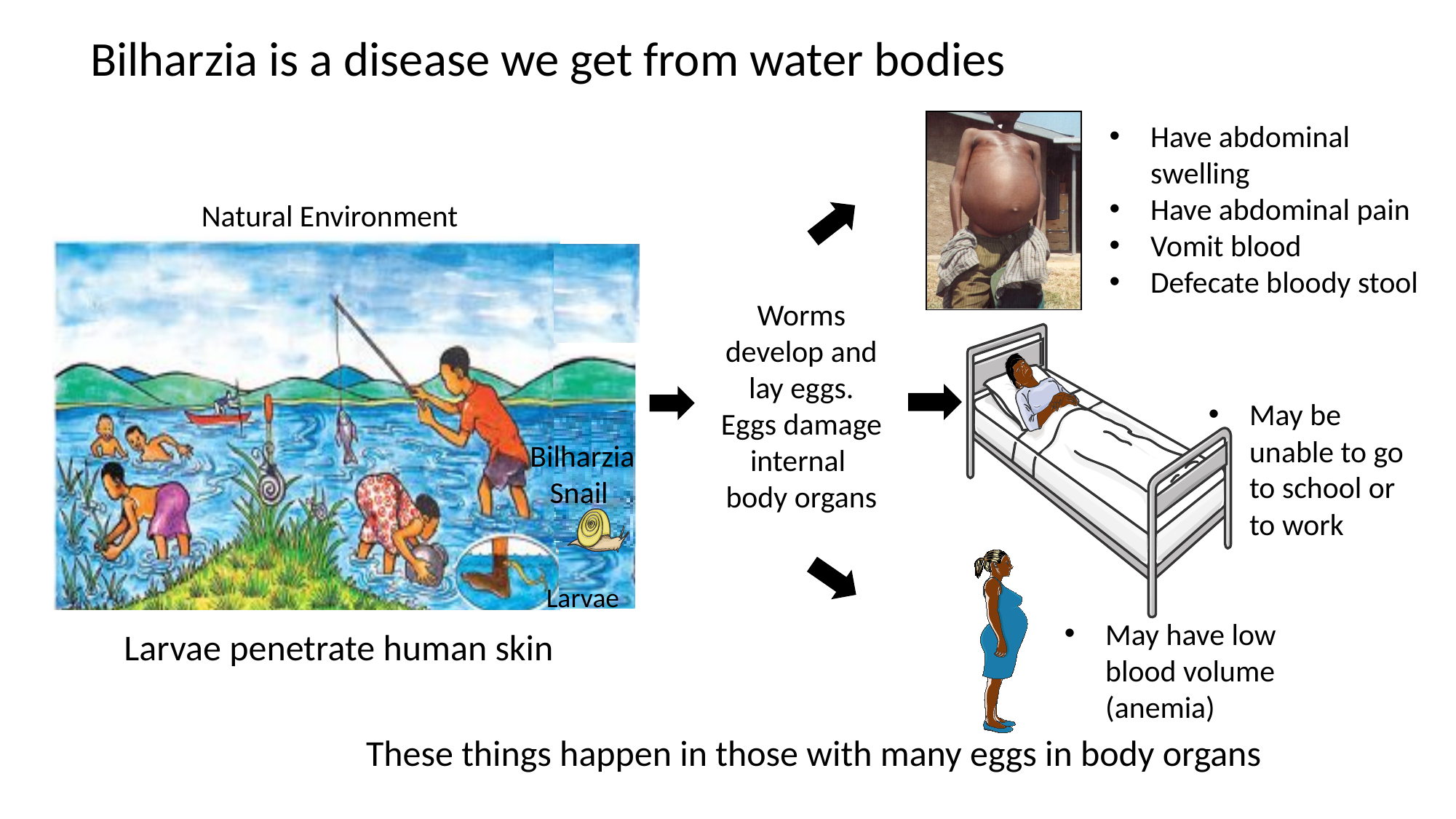

Bilharzia is a disease we get from water bodies
Have abdominal swelling
Have abdominal pain
Vomit blood
Defecate bloody stool
Natural Environment
Worms develop and lay eggs.
Eggs damage
internal
body organs
May be unable to go to school or to work
Bilharzia
Snail
Larvae
May have low blood volume (anemia)
Larvae penetrate human skin
These things happen in those with many eggs in body organs

## Slide 2
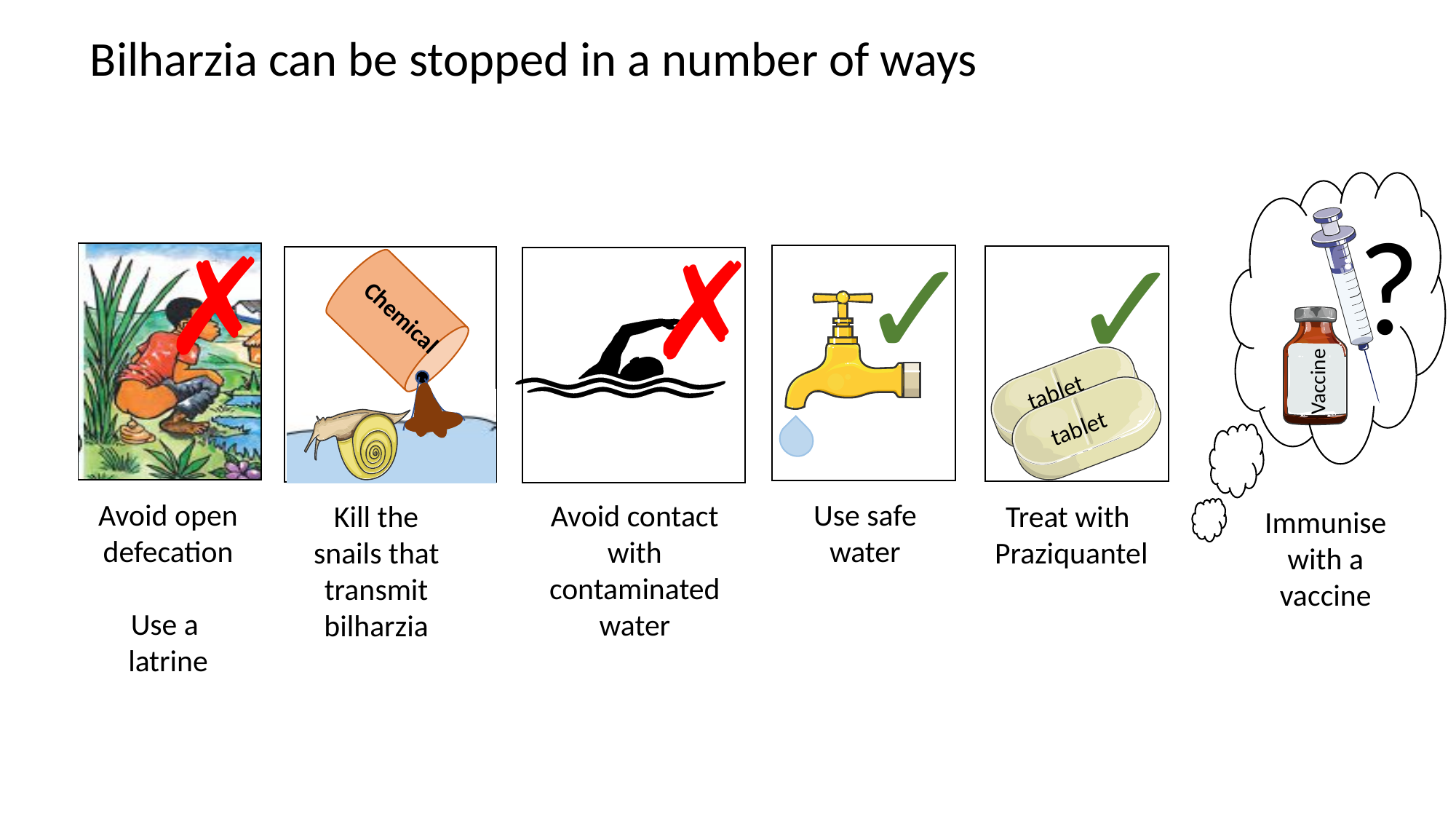

Bilharzia can be stopped in a number of ways
?
✗
✓
✓
✗
Chemical
Vaccine
tablet
tablet
Use safe water
Avoid open defecation
Use a
latrine
Avoid contact with contaminated water
Kill the snails that transmit bilharzia
Treat with
Praziquantel
Immunise with a vaccine

## Slide 3
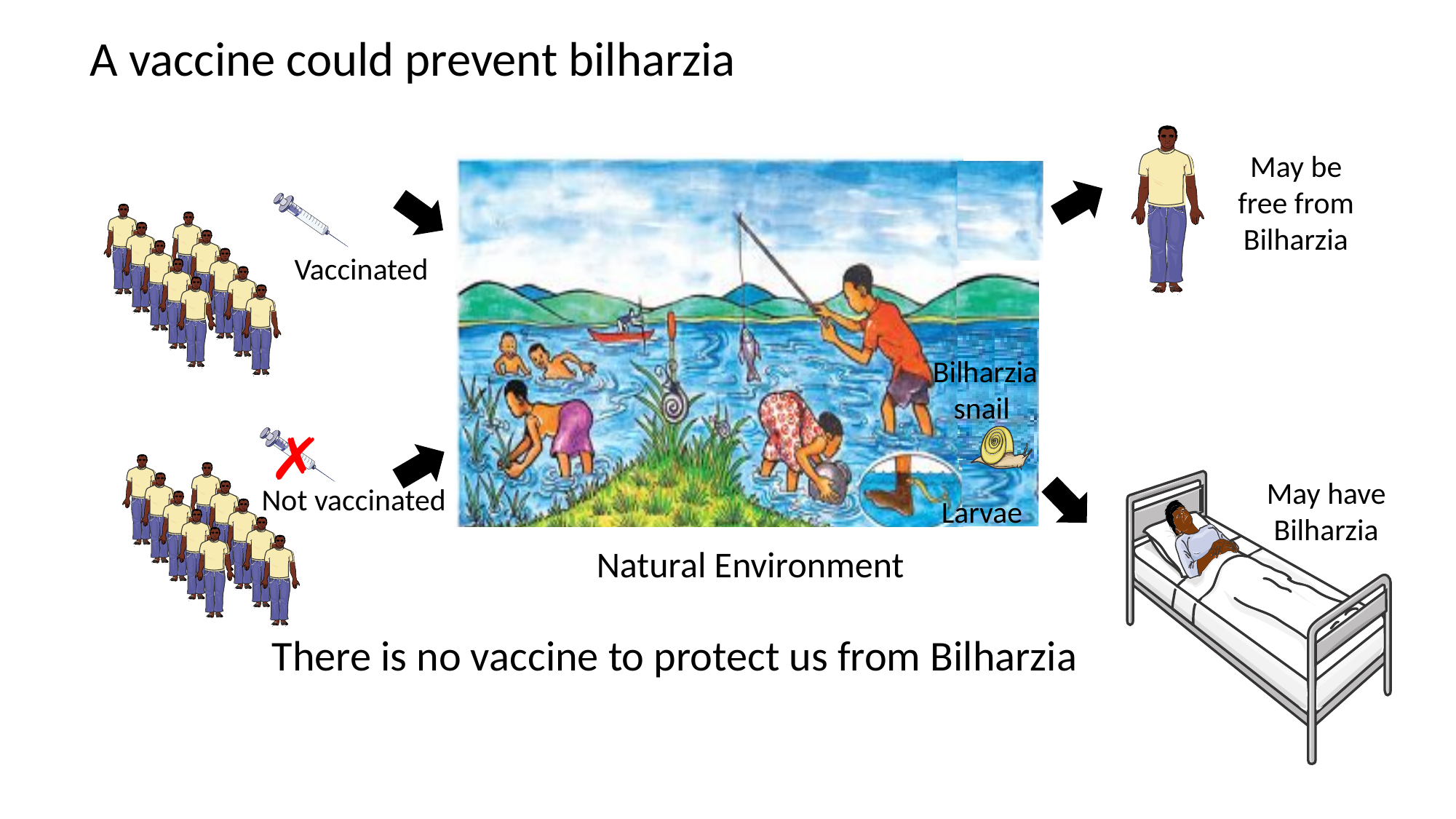

A vaccine could prevent bilharzia
May be free from Bilharzia
Vaccinated
Bilharzia
snail
✗
May have Bilharzia
Not vaccinated
Larvae
Natural Environment
There is no vaccine to protect us from Bilharzia

## Slide 4
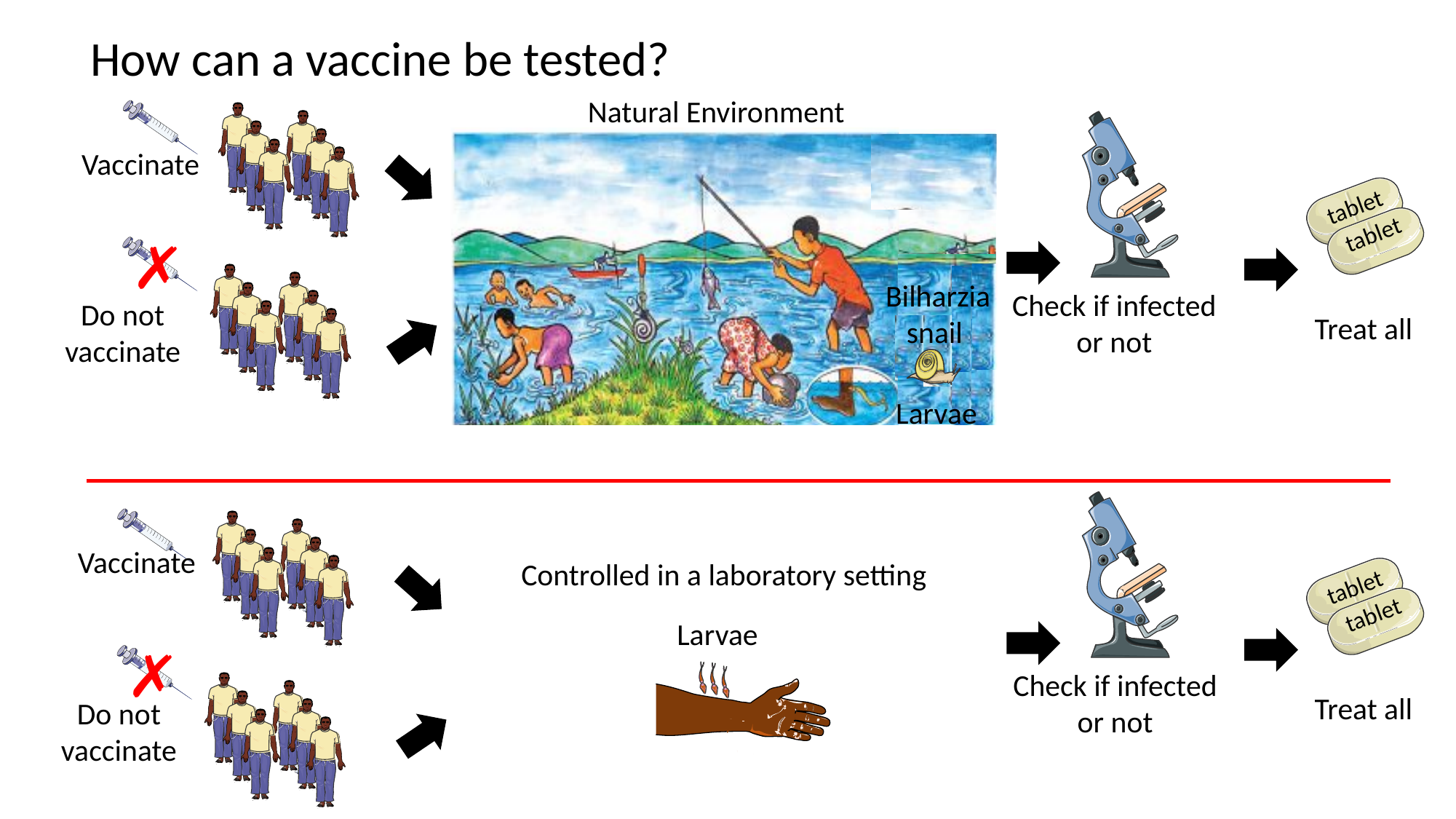

How can a vaccine be tested?
Natural Environment
Vaccinate
tablet
tablet
✗
Bilharzia
snail
Check if infected or not
Do not vaccinate
Treat all
Larvae
Vaccinate
Controlled in a laboratory setting
tablet
tablet
Larvae
✗
Check if infected or not
Treat all
Do not vaccinate

## Slide 5
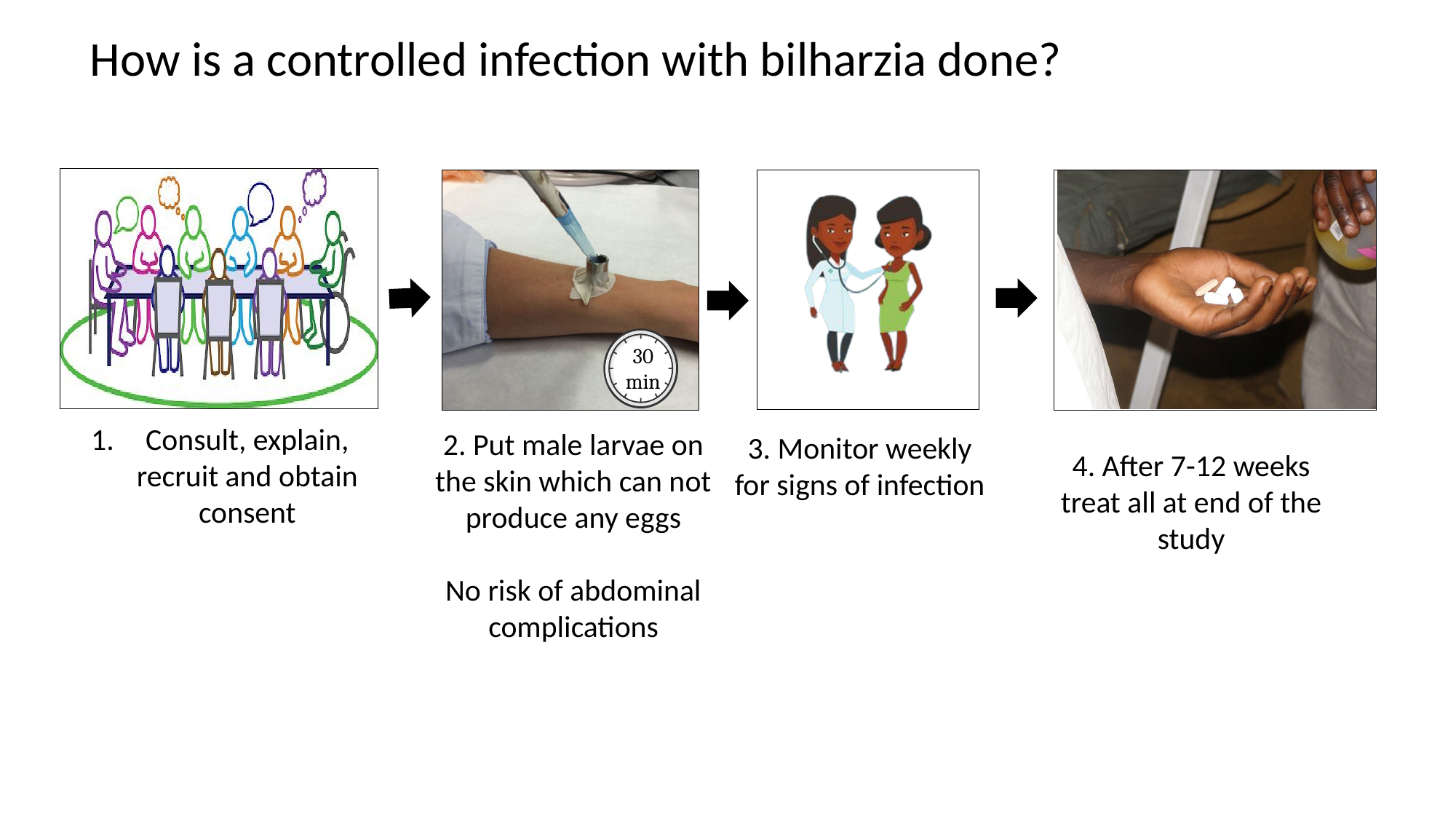

How is a controlled infection with bilharzia done?
30
min
Consult, explain, recruit and obtain consent
2. Put male larvae on the skin which can not produce any eggs
No risk of abdominal complications
3. Monitor weekly for signs of infection
4. After 7-12 weeks treat all at end of the study

## Slide 6
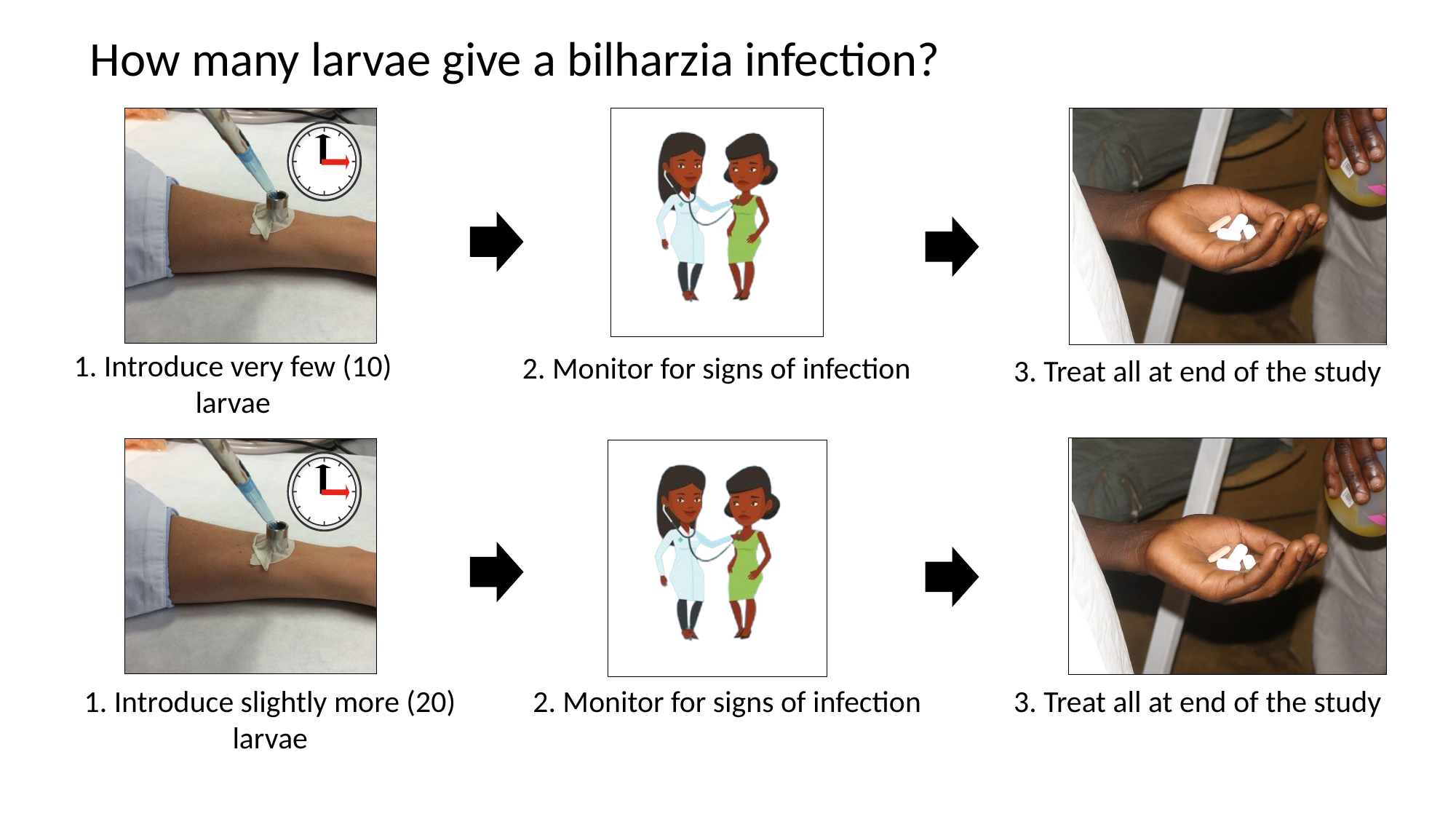

How many larvae give a bilharzia infection?
1. Introduce very few (10) larvae
2. Monitor for signs of infection
3. Treat all at end of the study
1. Introduce slightly more (20) larvae
2. Monitor for signs of infection
3. Treat all at end of the study

## Slide 7
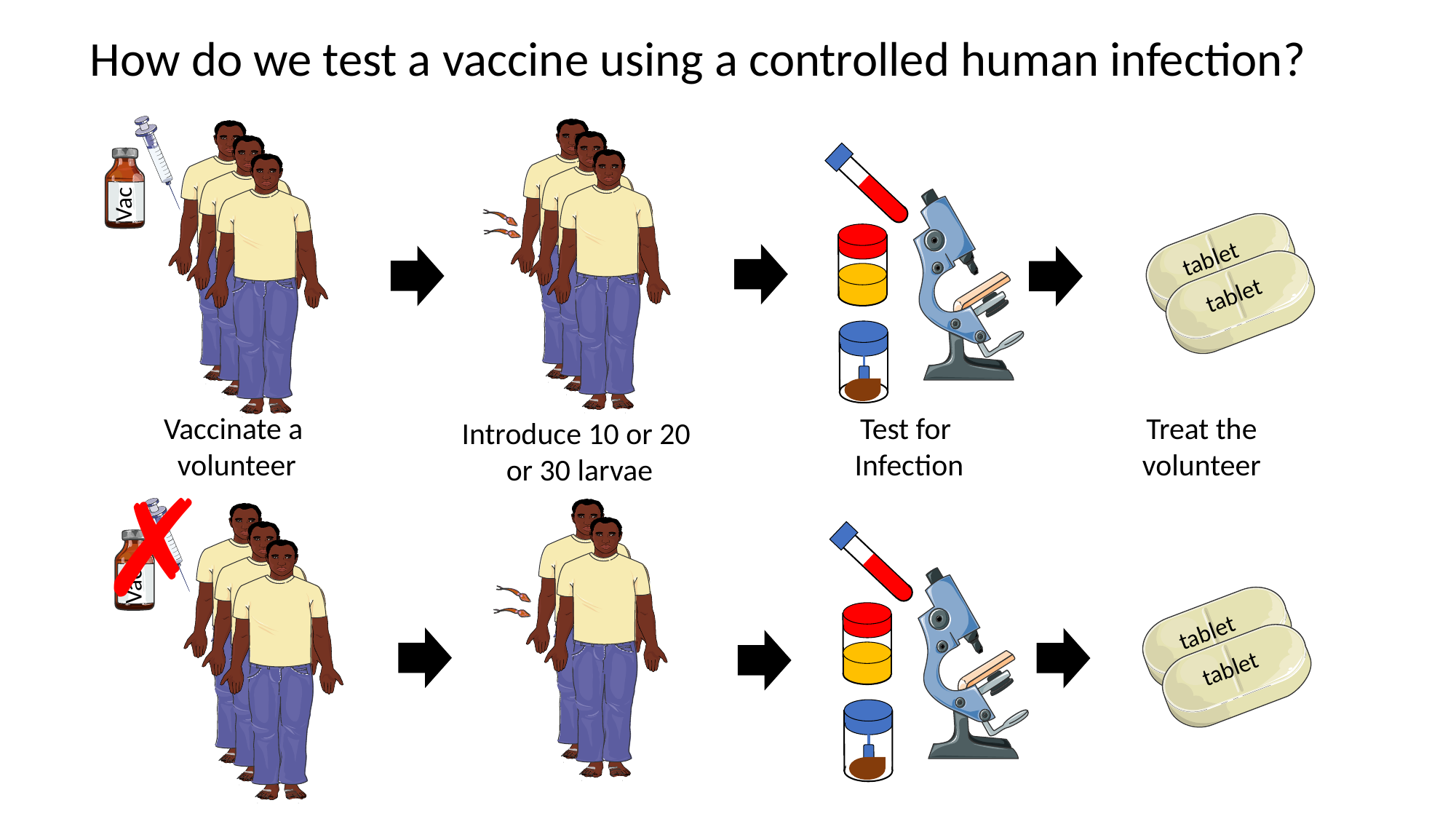

How do we test a vaccine using a controlled human infection?
Vac
tablet
tablet
Introduce 10 or 20
or 30 larvae
Treat the
volunteer
Vaccinate a
volunteer
Test for
Infection
✗
Vac
tablet
tablet

## Slide 8
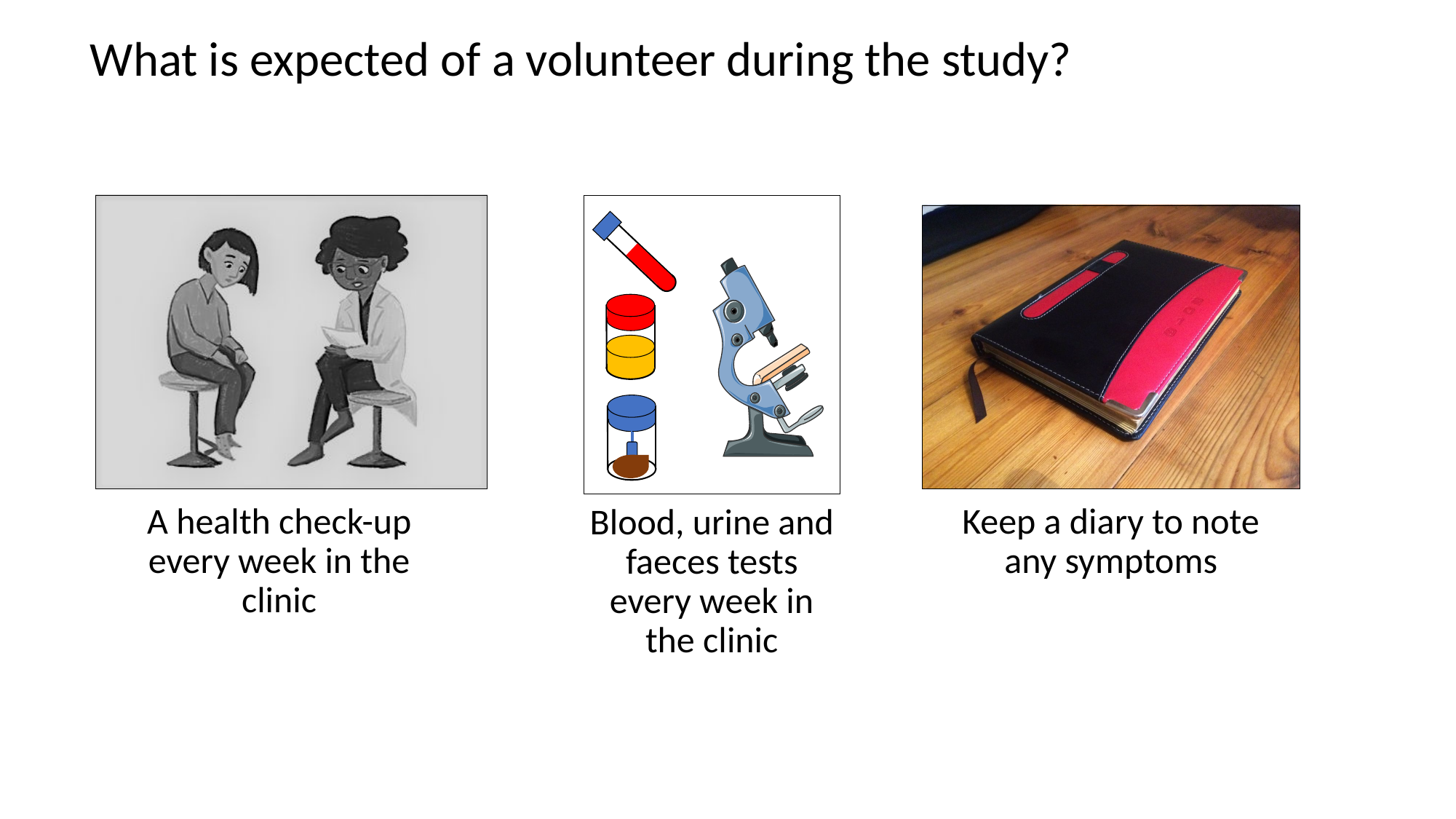

What is expected of a volunteer during the study?
A health check-up every week in the clinic
Keep a diary to note any symptoms
Blood, urine and faeces tests every week in the clinic

## Slide 9
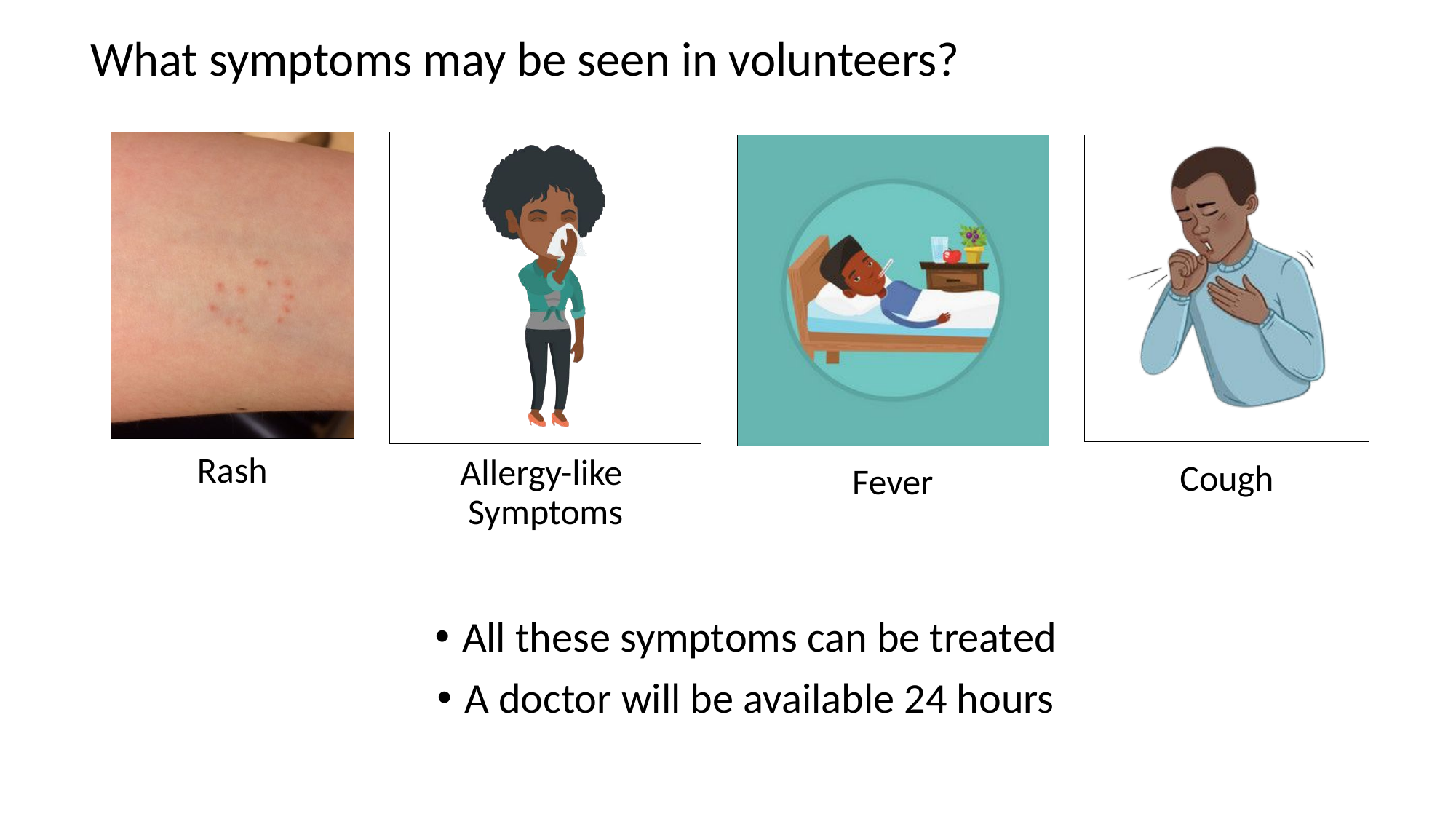

What symptoms may be seen in volunteers?
Rash
Allergy-like
Symptoms
Cough
Fever
All these symptoms can be treated
A doctor will be available 24 hours

## Slide 10
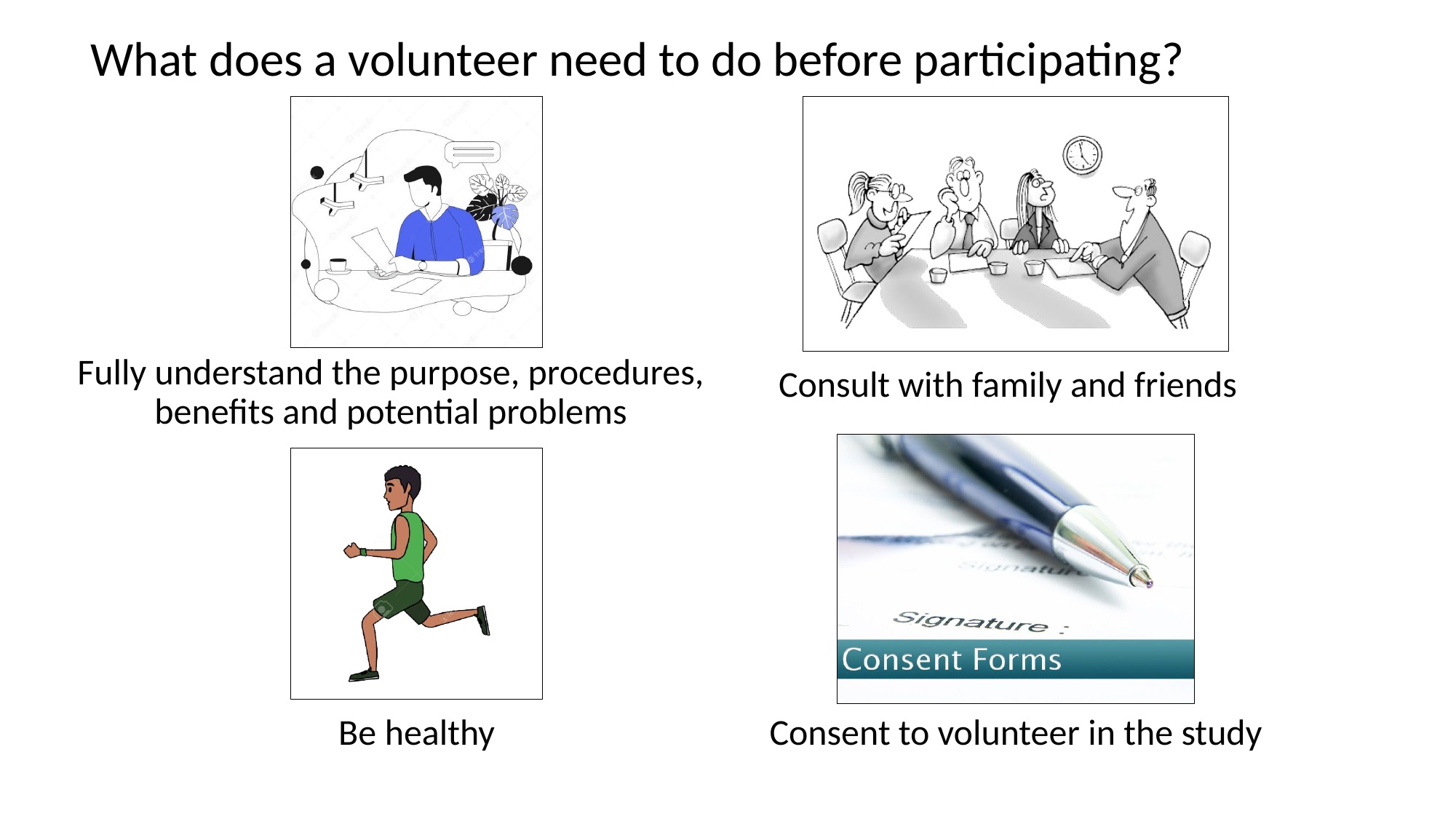

What does a volunteer need to do before participating?
Fully understand the purpose, procedures, benefits and potential problems
Consult with family and friends
Be healthy
Consent to volunteer in the study

## Slide 11
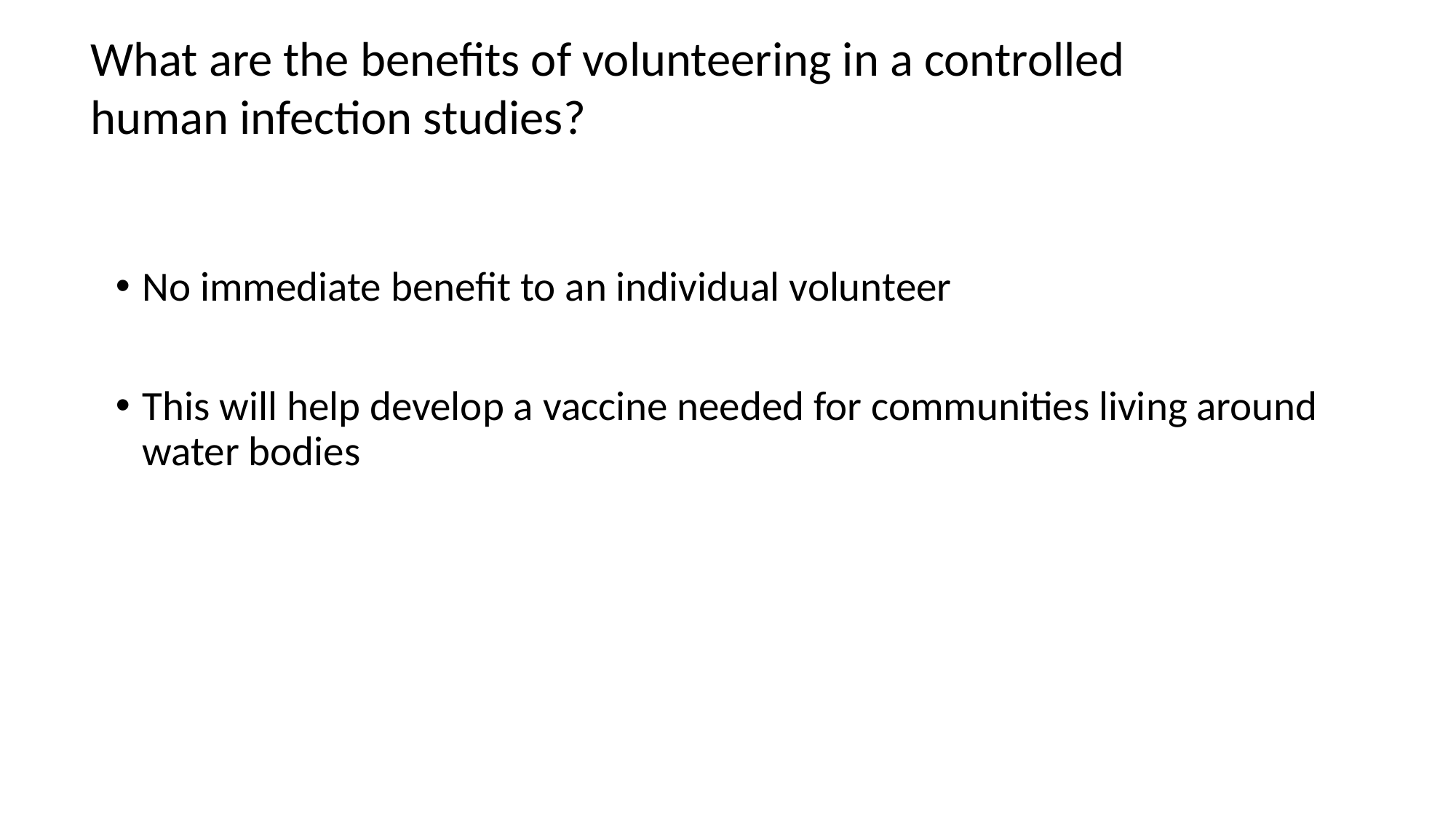

What are the benefits of volunteering in a controlled human infection studies?
No immediate benefit to an individual volunteer
This will help develop a vaccine needed for communities living around water bodies
