# Supplementary material for: Ethical and practical considerations arising from community consultation on implementing controlled human infection studies using Schistosoma mansoni in Uganda
Source: Glob Bioeth. 2022 Jul 4;33(1):78–102. doi: 10.1080/11287462.2022.2091503 (PMC9258062; doi:10.1080/11287462.2022.2091503)
Supplement: Supplemental Material [file RGBE_A_2091503_SM9810.doc]

**Supporting information**

**S1. Scientific details of the CHI-S**

**Medical importance, pathology and life cycle of *S. mansoni***

*Schistosoma mansoni* eggs retained in tissue provoke tissue inflammation, fibrosis, and portal hypertension (Grevelding, 2004). Adult *S. mansoni* worms live in the human portal blood vessels. Eggs excreted in stool hatch in water, each releasing a single miracidium which infects the intermediate *Biomphalaria* snail host where it reproduces asexually. Snails release thousands of cercariae, which can penetrate the human skin, transform into schistosomula, migrate into tissues, blood vessels and the lungs, and develop again as adults in the portal vessels, perpetuating the life cycle (figure 1).

*
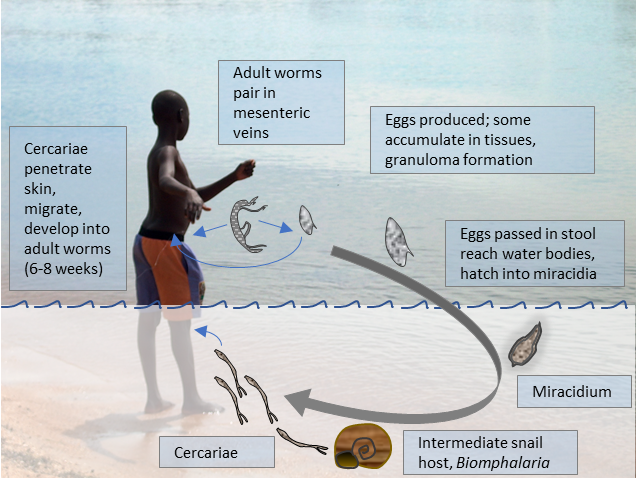
*

Figure 1. The life cycle of *Schistosoma mansoni* (Elliott et al., 2018).

**The need for a vaccine for schistosomiasis**

In high-transmission settings, mass drug administration (MDA) may fail to control transmission (Crellen et al., 2016; Gurarie et al., 2015). Our recent work among the fishing villages of the Lake Victoria island communities has shown that MDA can reduce *S. mansoni* infection intensity, but 85% of the population retained at least a light infection, even with quarterly treatment, representing a persistent reservoir for future reinfection (Sanya et al., 2019). Repeated cycles of praziquantel may lead to selection for relatively praziquantel resistant worm infections (Crellen et al., 2016). An effective vaccine would promote immunity to reinfection and circumvent drug resistance, and is therefore urgently needed (Alsallaq et al., 2017). Several *S. mansoni* (*Sm*) vaccines in preclinical and early clinical development (*Sm*14, *Sm*-TSP-2 and *Sm*p80) are potential candidates for efficacy testing in humans. Controlled human infections (CHI) can accelerate identification of the most promising vaccine candidates (Roestenberg et al., 2018; Sauerwein et al., 2011; Waddington et al., 2014). CHI studies utilize healthy volunteers but are usually followed by larger field vaccine efficacy trials where possible rare side effects may be identified. CHI studies provide benchmark efficacy endpoints for these efficacy trials (Chen et al., 2016) and identify candidate vaccines for which investment in Phase III trials is likely to be most rewarding.

**Controlled human infection with *S. mansoni***

In the laboratory, infecting individual laboratory snails (*Biomphalaria* species) with a single *Sm* miracidium results in a clone of single-sex cercariae. Male clones of cercariae can be identified by polymerase chain reaction and these are used for infection because adult males will not produce eggs, hence, egg-induced pathology in schistosomiasis is avoided (Janse et al., 2018). The controlled human infection with *S. mansoni* (CHI-S) involves applying a selected number of male *Sm* cercariae onto the human skin and the *Sm* cercariae naturally penetrate the skin. A team at the Leiden University Medical Center in the Netherlands has safely conducted the first CHI-S. In a proof-of-concept trial, approximately 80% of healthy individuals were successfully infected as measured with a highly-sensitive lateral flow assay for schistosome-specific circulating anodic antigen (CAA) in blood (Langenberg et al., 2020). In all cases infections were treated effectively using praziquantel.

**Transfer to *S. mansoni* endemic country**

Establishing the CHI-S model in Uganda, where *S. mansoni* is endemic has immunological considerations. Prior schistosome exposure will impact immune responses to *S. mansoni* infection and candidate vaccines: individuals can be sensitised to schistosomes prenatally and in very early childhood (Bustinduy et al., 2017; Novato-Silva et al., 1992). In addition, differences in co-infections and other environmental exposures are likely to result in important differences in vaccine response between Europe and tropical Africa (Muyanja et al., 2014). Establishing experimental infections in a previously exposed population will be highly informative in dissecting population differences in immune responses and will prepare Uganda for future accelerated testing of vaccine candidates.

**References**

Alsallaq, R. A., Gurarie, D., Ndeffo Mbah, M., Galvani, A., & King, C. (2017). Quantitative assessment of the impact of partially protective anti-schistosomiasis vaccines. *PLoS Neglected Tropical Diseases*, *11*(4), e0005544. https://doi.org/10.1371/journal.pntd.0005544

Bustinduy, A. L., Wright, S., Joekes, E. C., Kabatereine, N. B., Reinhard-Rupp, J., King, C. H., & Stothard, J. R. (2017). One hundred years of neglect in paediatric schistosomiasis. *Parasitology*, *144*(12), 1613–1623. https://doi.org/10.1017/S0031182017000014

Crellen, T., Walker, M., Lamberton, P. H. L., Kabatereine, N. B., Tukahebwa, E. M., Cotton, J. A., & Webster, J. P. (2016). Reduced Efficacy of Praziquantel Against Schistosoma mansoni Is Associated With Multiple Rounds of Mass Drug Administration. *Clinical Infectious Diseases : An Official Publication of the Infectious Diseases Society of America*, *63*(9), 1151–1159. PubMed. https://doi.org/10.1093/cid/ciw506

Elliott, A. M., Roestenberg, M., Wajja, A., Opio, C., Angumya, F., Adriko, M., Egesa, M., Gitome, S., Mfutso-Bengo, J., Bejon, P., Kapulu, M., Seager, Z., Lutalo, T., Nazziwa, W. B., Muwumuza, A., Yazdanbakhsh, M., Kaleebu, P., Kabatereine, N., & Tukahebwa, E. (2018). Ethical and scientific considerations on the establishment of a controlled human infection model for schistosomiasis in Uganda: Report of a stakeholders’ meeting held in Entebbe, Uganda. *AAS Open Research*, *1*, 2. https://doi.org/10.12688/aasopenres.12841.2

Grevelding, C. G. (2004). Schistosoma. *Current Biology*, *14*(14), R545. https://doi.org/10.1016/j.cub.2004.07.006

Gurarie, D., Yoon, N., Li, E., Ndeffo-Mbah, M., Durham, D., Phillips, A. E., Aurelio, H. O., Ferro, J., Galvani, A. P., & King, C. H. (2015). Modelling control of Schistosoma haematobium infection: Predictions of the long-term impact of mass drug administration in Africa. *Parasites & Vectors*, *8*, 529. https://doi.org/10.1186/s13071-015-1144-3

Janse, J. J., Langenberg, M. C. C., Kos-Van Oosterhoud, J., Ozir-Fazalalikhan, A., Brienen, E. A. T., Winkel, B. M. F., Erkens, M. A. A., van der Beek, M. T., van Lieshout, L., Smits, H. H., Webster, B. L., Zandvliet, M. L., Verbeek, R., Westra, I. M., Meij, P., Visser, L. G., van Diepen, A., Hokke, C. H., Yazdanbakhsh, M., & Roestenberg, M. (2018). Establishing the Production of Male Schistosoma mansoni Cercariae for a Controlled Human Infection Model. *The Journal of Infectious Diseases*, *218*(7), 1142–1146. https://doi.org/10.1093/infdis/jiy275

Langenberg, M. C. C., Hoogerwerf, M.-A., Koopman, J. P. R., Janse, J. J., Kos-van Oosterhoud, J., Feijt, C., Jochems, S. P., de Dood, C. J., van Schuijlenburg, R., Ozir-Fazalalikhan, A., Manurung, M. D., Sartono, E., van der Beek, M. T., Winkel, B. M. F., Verbeek-Menken, P. H., Stam, K. A., van Leeuwen, F. W. B., Meij, P., van Diepen, A., … Roestenberg, M. (2020). A controlled human Schistosoma mansoni infection model to advance novel drugs, vaccines and diagnostics. *Nature Medicine*, *26*(3), 326–332. PubMed. https://doi.org/10.1038/s41591-020-0759-x

Muyanja, E., Ssemaganda, A., Ngauv, P., Cubas, R., Perrin, H., Srinivasan, D., Canderan, G., Lawson, B., Kopycinski, J., Graham, A. S., Rowe, D. K., Smith, M. J., Isern, S., Michael, S., Silvestri, G., Vanderford, T. H., Castro, E., Pantaleo, G., Singer, J., … Gaucher, D. (2014). Immune activation alters cellular and humoral responses to yellow fever 17D vaccine. *The Journal of Clinical Investigation*, *124*(7), 3147–3158. https://doi.org/10.1172/JCI75429

Novato-Silva, E., Gazzinelli, G., & Colley, D. G. (1992). Immune responses during human schistosomiasis mansoni. XVIII. Immunologic status of pregnant women and their neonates. *Scandinavian Journal of Immunology*, *35*(4), 429–437. https://doi.org/10.1111/j.1365-3083.1992.tb02878.x

Roestenberg, M., Hoogerwerf, M.-A., Ferreira, D. M., Mordmüller, B., & Yazdanbakhsh, M. (2018). Experimental infection of human volunteers. *The Lancet. Infectious Diseases*, *18*(10), e312–e322. https://doi.org/10.1016/S1473-3099(18)30177-4

Sanya, R. E., Nkurunungi, G., Hoek Spaans, R., Nampijja, M., O’Hara, G., Kizindo, R., Oduru, G., Kabuubi Nakawungu, P., Niwagaba, E., Abayo, E., Kabagenyi, J., Zziwa, C., Tumusiime, J., Nakazibwe, E., Kaweesa, J., Muwonge Kakooza, F., Akello, M., Lubyayi, L., Verweij, J., … LaVIISWA Trial Team. (2019). The Impact of Intensive Versus Standard Anthelminthic Treatment on Allergy-related Outcomes, Helminth Infection Intensity, and Helminth-related Morbidity in Lake Victoria Fishing Communities, Uganda: Results From the LaVIISWA Cluster-randomized Trial. *Clinical Infectious Diseases : An Official Publication of the Infectious Diseases Society of America*, *68*(10), 1665–1674. PubMed. https://doi.org/10.1093/cid/ciy761

Sauerwein, R. W., Roestenberg, M., & Moorthy, V. S. (2011). Experimental human challenge infections can accelerate clinical malaria vaccine development. *Nature Reviews. Immunology*, *11*(1), 57–64. https://doi.org/10.1038/nri2902

Waddington, C. S., Darton, T. C., Woodward, W. E., Angus, B., Levine, M. M., & Pollard, A. J. (2014). Advancing the management and control of typhoid fever: A review of the historical role of human challenge studies. *The Journal of Infection*, *68*(5), 405–418. https://doi.org/10.1016/j.jinf.2014.01.006
